# Genetic and evolution analysis of extrafloral nectary in cotton

**DOI:** 10.1111/pbi.13366

**Published:** 2020-03-10

**Authors:** Wei Hu, Wenqiang Qin, Yuying Jin, Peng Wang, Qingdi Yan, Fuguang Li, Zhaoen Yang

**Affiliations:** ^1^ Zhengzhou Research Base, State Key Laboratory of Cotton Biology Zhengzhou University Zhengzhou China; ^2^ State Key Laboratory of Cotton Biology Institute of Cotton Research of the Chinese Academy of Agricultural Sciences Anyang China

**Keywords:** *Gossypium arboreum*, population, *F*_ST_, nectar, cell wall, jasmonic acid

## Abstract

Extrafloral nectaries are a defence trait that plays important roles in plant–animal interactions. *Gossypium* species are characterized by cellular grooves in leaf midribs that secret large amounts of nectar. Here, with a panel of 215 *G. arboreum* accessions, we compared extrafloral nectaries to nectariless accessions to identify a region of Chr12 that showed strong differentiation and overlapped with signals from GWAS of nectaries. Fine mapping of an F_2_ population identified *GaNEC1*, encoding a PB1 domain‐containing protein, as a positive regulator of nectary formation. An InDel, encoding a five amino acid deletion, together with a nonsynonymous substitution, was predicted to cause 3D structural changes in *GaNEC1* protein that could confer the nectariless phenotype. mRNA‐Seq analysis showed that JA‐related genes are up‐regulated and cell wall‐related genes are down‐regulated in the nectary. Silencing of *GaNEC1* led to a smaller size of foliar nectary phenotype. Metabolomics analysis identified more than 400 metabolites in nectar, including expected saccharides and amino acids. The identification of *GaNEC1* helps establish the network regulating nectary formation and nectar secretion, and has implications for understanding the production of secondary metabolites in nectar. Our results will deepen our understanding of plant–mutualism co‐evolution and interactions, and will enable utilization of a plant defence trait in cotton breeding efforts.

## Introduction

The extrafloral nectary (EFN) plays significant roles in plant defence against herbivores by enabling mutualisms with ants and other carnivorous insects that increase the fitness of both partners involved (Gish *et al.*, 2016; Yamawo *et al.*, 2019). In the mutualistic interaction, plants offer extrafloral nectar as a food resources to predatory ant partners and ants offer direct defence of nectary‐bearing plant parts against other insects (Heil *et al.*, 2001). It has been shown that ants protect plants and decrease herbivory, with positive contributions to the fitness of several plant species (Oliveira and Freitas, 2004). For example, the nectary‐bearing plant *Mallotus japonicas* used different types of EFNs to recruit ants to established effective defence against herbivores (Yamawo *et al.*, 2019). Herbivory, artificial damage and application of exogenous jasmonic acid (JA) can strongly induce EFN secretion, and inhibition of endogenous JA synthesis can block damage‐induced reactions (Heil *et al.*, 2001).

The evolutionary origins, morphologies and locations of EFNs on plants are diverse (Marazzi *et al.*, 2013). In more 100 angiosperm families, EFNs attract ants, generalist predators and parasitoids and provide indirect defence against herbivores (World List of Plants with Extrafloral Nectaries. www.extrafloralnectaries.org; Heil, 2011). Types of EFNs include extranuptial nectaries, circumfloral nectaries, postfloral nectaries and foliar nectaries can be located on every plant organ except roots (Heil, 2011; Nepi, 2007). EFNs are most abundant in the legume family (Fabaceae), Euphorbiaceae and Malvaceae, accounting for more than 40% of ENF‐bearing species (World List of Plants with Extrafloral Nectaries). Despite the EFN diversity, nectar composition is similar in different plant species (Marazzi *et al.*, 2013). Sugars and amino acids are well‐established nectar components, though new substances continue to be discovered (Nepi, 2007; Roy *et al.*, 2017).

*Gossypium*, a genus of Malvaceae, is comprised of more than 50 species (Wendel *et al.*, 2012; Yang *et al.*, 2019), most of which have nectaries in leaf midribs. Extrafloral nectar secretion can be induced by herbivores, and cotton EFNs may play a role in plant–mutualism indirect defence (Wackers and Bonifay, 2004; Wackers *et al.*, 2001). However, several studies have shown that cotton nectaries tend to attract agricultural pests, resulting in crop yield loss (Henneberry *et al.*, 1977; Lukefahr *et al.*, 1966; Lukefahr *et al.*, 1965; Lukefahr and Rhyne, 1960; Meredith *et al.*, 1973; Schuster *et al.*, 1976; Thomson *et al.*, 1987; Wilson and Wilson, 1976). Recent studies have shown that domestication of cotton has affected utilization of EFNs in indirect defence (Llandres *et al.*, 2019). Genetic analysis showed that a pair of recessive genes, located on homologous chromosomes A12 and D12, is responsible for nectary formation in allotetraploid cotton (Hou *et al.*, 2013; Meyer and Meyer, 1961; Waghmare *et al.*, 2005). Very recently, a comprehensive study of key metabolic modules of floral and extrafloral nectaries supports the coordination of merocrine‐based and eccrine‐based models of nectar synthesis in *G. hirsutum* (Chatt *et al*, 2019).

Despite the importance of EFNs, genetic understanding of EFN formation is underdeveloped. Investigations of the molecular genetic basis of nectary formation could provide environmental‐friendly options for developing pest resistance cultivars and improve understanding of plant‐animal co‐evolution. However, genes responsible for foliar nectaries, the regulation network for nectary formation and components of cotton nectary remain unknown. Comparing accessions bearing EFNs with those lacking EFNs in a panel of 215 *G. arboreum* accessions (Du *et al.*, 2018; Gong *et al.*, 2018), we found significant chromosomal differentiation in a region of Chr12 that overlaps a region identified by GWAS of the nectary trait and mapping of QTLs. With a mapping‐based cloning approach, we identify *GaNEC1* as the likely causative gene for nectaries in *G. arboreum*. mRNA‐seq showed that JA and cell wall‐related genes may play important roles in nectary development and nectar secretion. With a diversity expanded beyond carbohydrate and amino acid components, more than 400 metabolites were identified in nectar, which included an enrichment of phenylpropanoid synthesis metabolites. Uncovered molecular details about nectary formation could be applied to molecular breeding of cultivars that confer pest resistance via biological control.

## Results

### Origin and structure of EFNs in cotton

**Figure 1:**
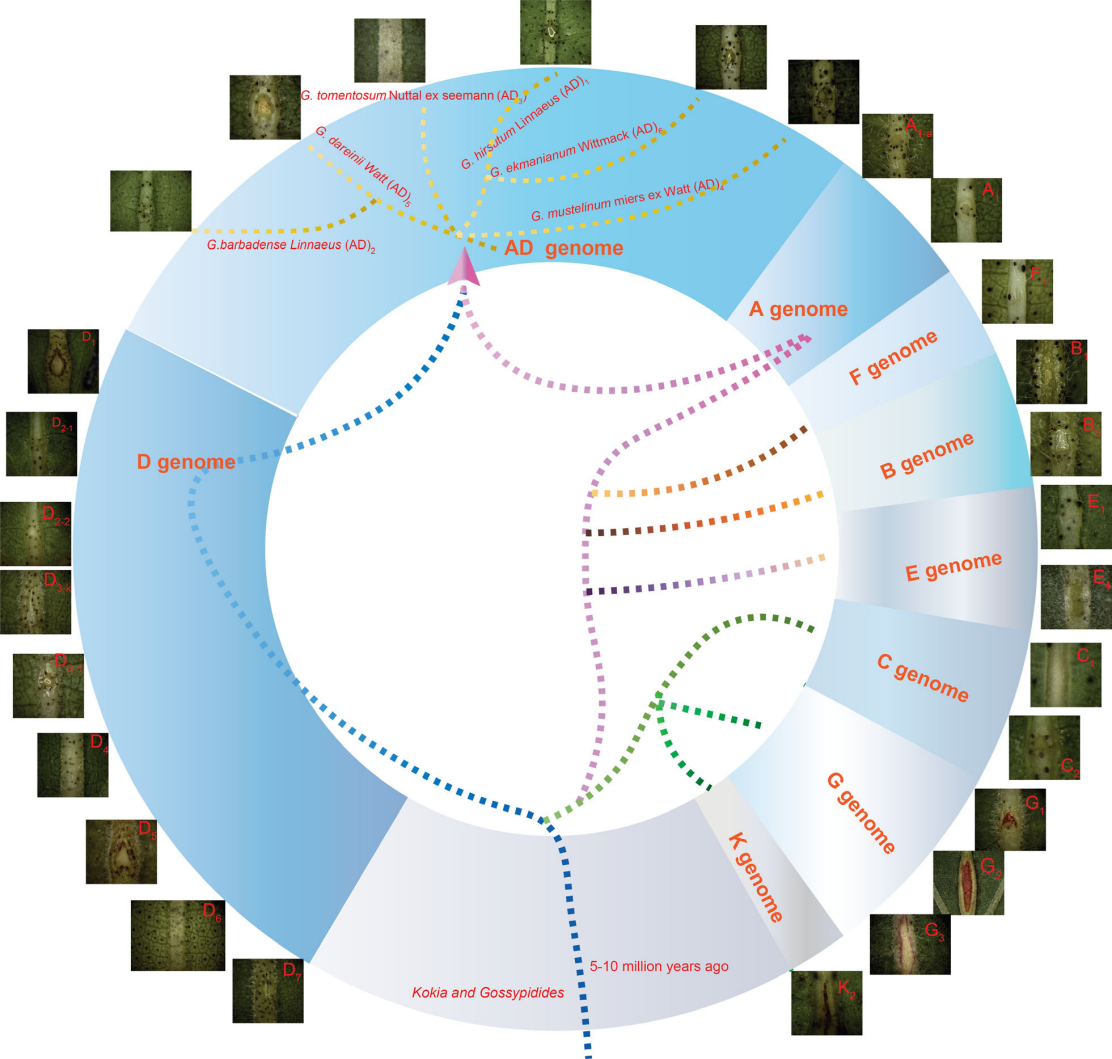
Investigation of foliar nectary in 27 diverse cotton species. The 27 species belong to 8 chromosomal groups (A to G plus K) and allotetraploid AD group. *G. gossypiodes* (Ulbrich) Standley (D6) and allotetraploid *G. tomentosum* (AD_3_) did not have any nectaries on the midribs of leaves.

We checked for foliar nectaries in 27 cotton species that belong to the eight cotton chromosome groups (A through G, plus K) and the AD allotetraploid group. All of the investigated species had EFNs, except for the diploid cotton *G. gossypiodes* (Ulbrich) Standley (D_6_) and allotetraploid *G. tomentosum* (AD_3_) (Figure 1 and Table S1). Molecular phylogenetic analysis suggested that the *Gossypium* genus diverged 5–10 million years ago from Hawaiian *Kokia* and the African–Madagascaran genus *Gossypioides* (Wendel *et al.*, 2010). Previous studies indicated that these two species contain nectaries on the underside of leaf midribs (Keeler, 1985; Luteyn and Fryxell, 1980). Taken together, our results indicate that the EFNs of *Gossypium* spp. originated in common ancestors *Kokia* and *Gossypioides*, and the foliar nectary variety developed via natural mutation during *Gossypium* divergence.

**Figure 2:**
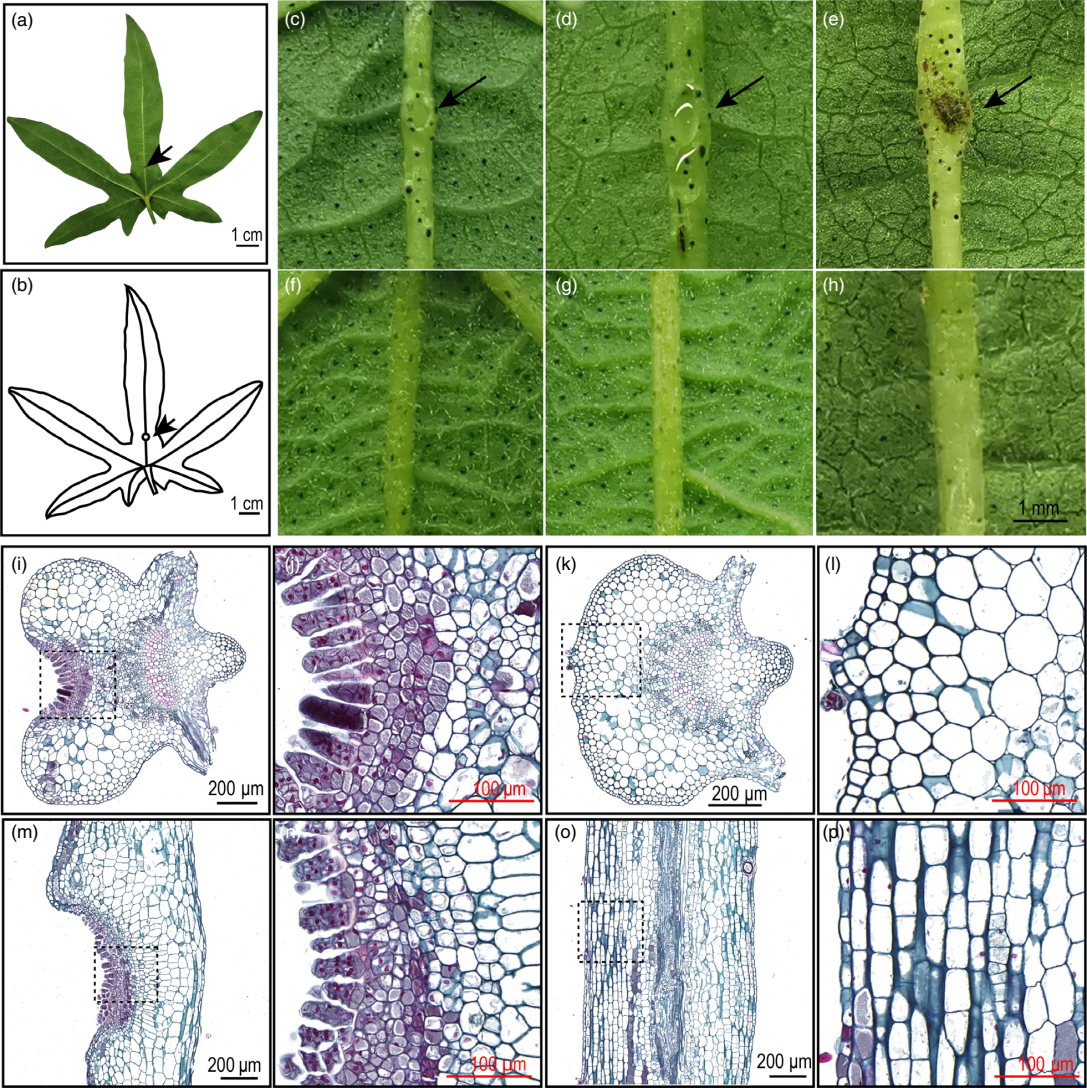
Phenotype of foliar nectary and nectariless. (a) Adult leaf bearing a nectary. (b) Diagram of nectary location in the leaf midrib. (c) Nectary of a young leaf. (d) Nectary of an adult leaf with strong nectar secretion. (e) Nectary of an old leaf. (f‐g) The phenotypes of nectariless leaves at young, adult and old leaf stages. (i) Microstructure of the nectary in a transverse section. (j) Enlargement of the dotted line box in (i). (k) Microstructure of nectariless tissue in a transverse section. (j) Enlargement of the dotted line box in (k). (m) Microstructure of the nectary in a longitudinal section. (j) Enlargement of the dotted line box in (m). (o) Microstructure of nectariless tissue in a longitudinal section. (p) Enlargement of the dotted line box in (o). The arrows indicate nectary locations.

We also observed the foliar nectariless trait is widely distributed in a collection of 215 *G. arboreum* landraces that had been widely cultivated in China from the Song dynasty to the 1950s (Du *et al.*, 2018). *G. arboreum* displayed EFNs, which is a concave structure about 1 cm from the leaf base, that are located on the undersides of leaves along the midrib (Figure 2a‐b). The nectary development in different accessions showed variable timing, with some species, like *G. hirsutum* acc. TM‐1, bearing EFNs in the cotyledon essentially at emergence, while other species, like the *G. arboreum,* form EFNs at the true leaf stage (Figure S1). The external morphology of the *G. arboreum* EFN changed during leaf development. We observed that young leaves have a smaller nectary that secretes less nectar, adult leaves have a larger nectary that secretes substantial nectar and EFNs on old leaves appear to lose secretion function and turn black (Figure 2c‐e). Nectariless accessions did not grow any concave structures on the midrib during leaf maturation and senescence (Figure 2f‐h).

Ultrastructure analysis showed that secretory trichomes, which serve as secretory structures, are located in the nectary epidermis and form an arc line structure (Figure 2i‐j). In contrast, identifiable differentiated tissues were not observed in both transverse and longitude sections of nectariless mid‐veins (Figure 2k, i, o and p). We observed that the secretory trichomes are multicellular, capitate trichomes (Figure 2j, n and Figure S2) that are like those in the EFNs of *Vicia faba* and *G. hirsutum* (Chatt *et al.*, 2019; Davis *et al.*, 1988). Beneath the epidermis, we observed five layers of small cells with densely stained cytoplasm, which are involved in pre‐nectar transport of nectar carbohydrates. The nectary parenchyma showed irregular polygon shapes in both transverse and longitudinal sections (Figure 2j and n). The cells underlying the epidermis were larger and elliptical in transverse sections of foliar nectariless midribs and rectangular in longitudinal ones (Figure 2i and p). Thus, it is clear that the morphological structures of cotton nectary are quite different from those in nectariless leaves that do not contain secretory cells and structures.

### Natural selection of nectariless in improvements efforts

**Figure 3:**
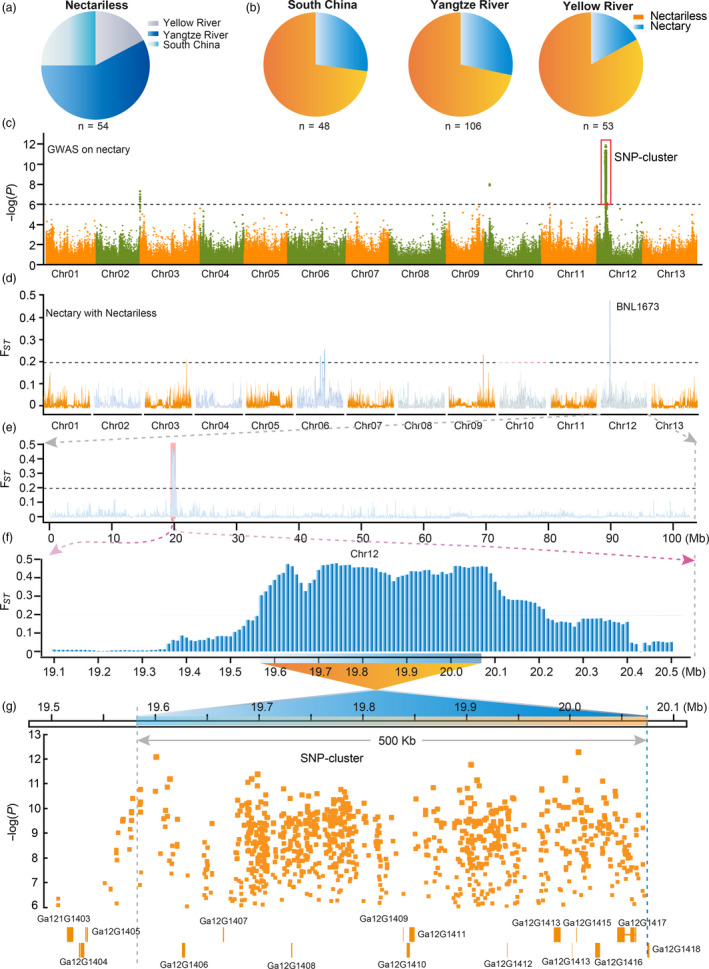
Both *F*_ST_ and GWAS analysis identified the same region in *G. arboreum* genome as a potential location for the nectariless trait. (a) The geographical distribution of accessions without foliar nectaries. (b) The distribution of nectariless frequency in different geographical populations of *G. arboreum*. Nectary and nectariless are shown in blue and orange, respectively. (c), GWAS examining nectary in a *G. arboreum* population. The strongest associated SNPs (SNP cluster) are marked by red boxes with the black dashed line indicating the threshold for GWAS signals. (d) *F*_ST_ analysis across whole genome between nectary and nectariless groups. Black dashes indicate the threshold for *F*_ST_. (e) A region of Chr12 showing the strongest selective signal. (f) Enlargement of the selective signal in (e). (g) GWAS results overlap with the *F*_ST_ region of Chr12. The orange boxes represent candidate genes, and orange dots are the associated SNPs in the GWAS signal region. Ga12G1403‐1418 are gene loci.

Population genetics results suggested that the Chinese *G. arboreum* can be classified as three geographical–ecological types: South China (SC), Yangtze River (YZR) and Yellow River (YER) (Table S2; Du *et al.*, 2018). We found 161 accessions bear EFNs and 54 accessions do not develop EFNs in the foliar vein (Figure 3a). Thus, it seems that nectariless accessions were widely selected by early agricultural production in China. Further inspection of the distribution of these nectariless accessions revealed that most of them are from the SC and YZR groups, and the frequency of the nectariless trait in YZR and SC groups was more than 10% higher than that of the YER group (Figure 3b). These results indicate that the EFN trait has been selected over a diverse agronomy–geography.

Next, we performed GWAS of nectary and found a strong association signal on chromosome 12 (~19.5 to ~20.1 Mb; Figure 3c). This genetic information reflects human efforts to reshape agronomic traits. We then analysed the population differentiation (*F*_ST_) of nectary and nectariless groups using VCFtools with a 50 kb sliding window and 10 kb step (Danecek *et al.*, 2011). We found improvement sweeps on Chr06, Chr09 and Chr12 (Figure 3d). Strikingly, the sweep region on Chr12 overlapped with the strong association signal on Chr12, indicating that the nectariless trait was artificially selected during improvement. Zooming‐in on the differentiated signal on Chr12 revealed a ~0.64 Mb region (19.57 to 20.21 Mb) that displayed significant population differentiation with an average *F*_ST_ equal to 0.40 compared to 7.8 × 10^−3^ measured at the whole‐genome scale (Figure 3e‐f). A SNP cluster on Chr12 spans from 19.55 to 20.07 Mb and shares 500 kb (19.57–20.07 Mb) of overlap with the *F*_ST_ region, identifying the 500 kb segment as an important candidate region for a nectariless gene. Eleven genes are located in the 500 kb region, among which *Ga12G1408*, *Ga12G1409* and *Ga12G1412* bear SNPs in exons and *Ga12G1413*, *Ga12G1416* and *Ga12G1417* have SNPs in introns (Figure 3g). These represented candidate genes for the nectariless trait, and fine mapping was pursued to identify a true, causal gene.

### Bulk segregation analysis and fine mapping of EFNs

**Figure 4:**
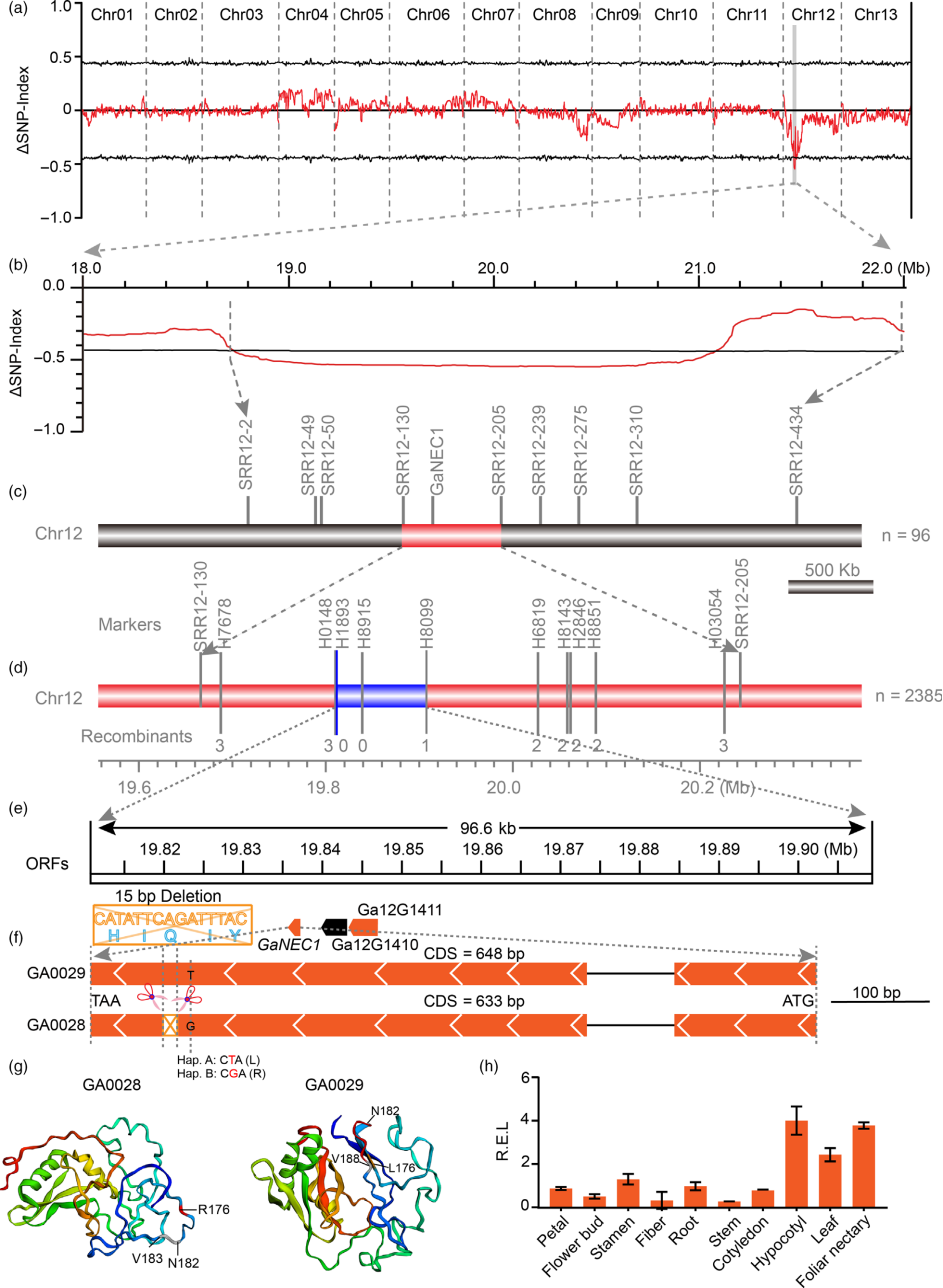
BSA analysis and fine mapping of nectariless. (a) QTL analysis of the nectariless phenotype in an F_2_ segregating population. The ∆SNP index (SNP index of the nectariless bulk population subtracted from that of the nectary bulk population) and its 99% confidence interval are shown as red and black lines, respectively. (b) Enlargement of the QTL signal in (a). (c‐e) Fine mapping of nectariless with SSR and InDel primers. Three ORFs located in the 96.6 kb candidate region are indicated by wedge boxes. (f) A 15‐bp InDel and one nonsynonymous mutation in the second exon of *GaNEC1* resulted in a five amino (HIQIY) deletion and a Leu (L)‐to‐Arg (R) substitution, respectively, in the putative protein sequence. (g) Protein structure modelling of GaNEC1. N182 and V183 (grey) are the amino acids adjacent to the 5 amino acids deleted in GA0028. The site of deleted p.H183/p.I184/p.185/p.I186/p.187 is marked in red between the p.N182 and p.V188. The nonsynonymous mutation in 176 AA was marked in red. (h) *GaNEC1* expression in different tissues. The data in (h) represent the means ± SE from three independent experiments.

To confirm the GWAS result and identify causal genes for EFNs, an EFN‐bearing accession GA0029 (JiangSuHongJingJiJiao) and a non‐EFN accession GA0028 (Xiaobaihua) from the GWAS population were selected as parents for construction of an F_2_ population through hybridization and selfing. All F_1_ plants had foliar nectaries on the underside of midribs, indicating that the nectariless allele is recessive. The F_2_ population produced a 1:3 segregation ratio of non‐EFN to EFN phenotypes (Table S3), indicating that a single locus controlled nectary formation. Next, we used a bulk segregation analysis (BSA) strategy to identify the potential nectary locus (Figure 4a). As expected, the ΔSNP index greater than 99% confidence intervals (QTLs region, *nec1* locus) from BSA was also located on chromosome 12 (Figure 4a). Detailed examination of QTLs led us to a 2.34 Mb region (18.74–21.08 Mb) on Chr12 that contains the 500 kb candidate region was identified by GWAS combined with *F*_ST_, further confirming that a gene on Chr12 controls the nectary trait (Figure 4b). Linkage analysis using a population of 96 F_2_ individuals confirmed that QTLs from Chr12 controls EFNs and narrowed focus to a putative causal gene, *GaNEC1*, located in the 576 kb segment between molecular markers SSR12‐22 (19 666 348 bp) and SSR12‐205 (20 243 100 bp) (Figure 4c). Next, we encrypted the density of molecular markers in the 576 kb region on the basis of InDels between the two GA0028 and GA0029 parents. A total of 297 markers were designed, and 15 markers had PCR‐confirmed polymorphisms between the two parents that were used for fine mapping in a large F_2_ population (Table S4). Finally, the nectary locus (*nec1*) was narrowed down to a 96.6 kb region that spans three annotated genes and is flanked by H0148 and H8099 (Figure 4d‐e). The three annotated genes encode a Phox and Bem1 (PB1) domain gene (*Ga12G1409*), a Myb_DNA‐bind_3 domain (*Ga12G1410*) and a gene with unknown function (*Ga12G1411*).

We cloned and sequenced these putative ORFs from genomic DNA and found that there were no differences between the two mapping parents in the *Ga12G1410* and *Ga12G1411* ORFs (Figure S3). In contrast, there were two sequence differences in *GaNEC1* from GA0028 compared to that in GA0029: a 15‐bp deletion was observed at bp 547 to bp 561 in the coding sequence (CDS), which resulted in a five amino (HIQIY) deletion, and a SNP (T/G) at position 530 in the CDS, which results in a leucine (L)‐to‐arginine (R) substitution (Figure 4f). Protein modelling with Phypre2 at 90% accuracy indicated that the five deleted amino acids formed a coil and the nonsynonymous substitution is located in a β‐sheet. The combined mutations are predicted to result in the disappearance of two β‐sheets and a complete change in the 3D structure of GaNEC1 (Figure 4g), consistent with the two alleles of *GaNEC1* having diverse functions. Expression analysis showed that *GaNEC*1 was highly expressed in hypocotyl and foliar nectary, consistent with a role for *GaNEC1* in nectary formation (Figures 4h and S4).

### Differentially expressed genes between EFN and non‐EFNs plants

**Figure 5:**
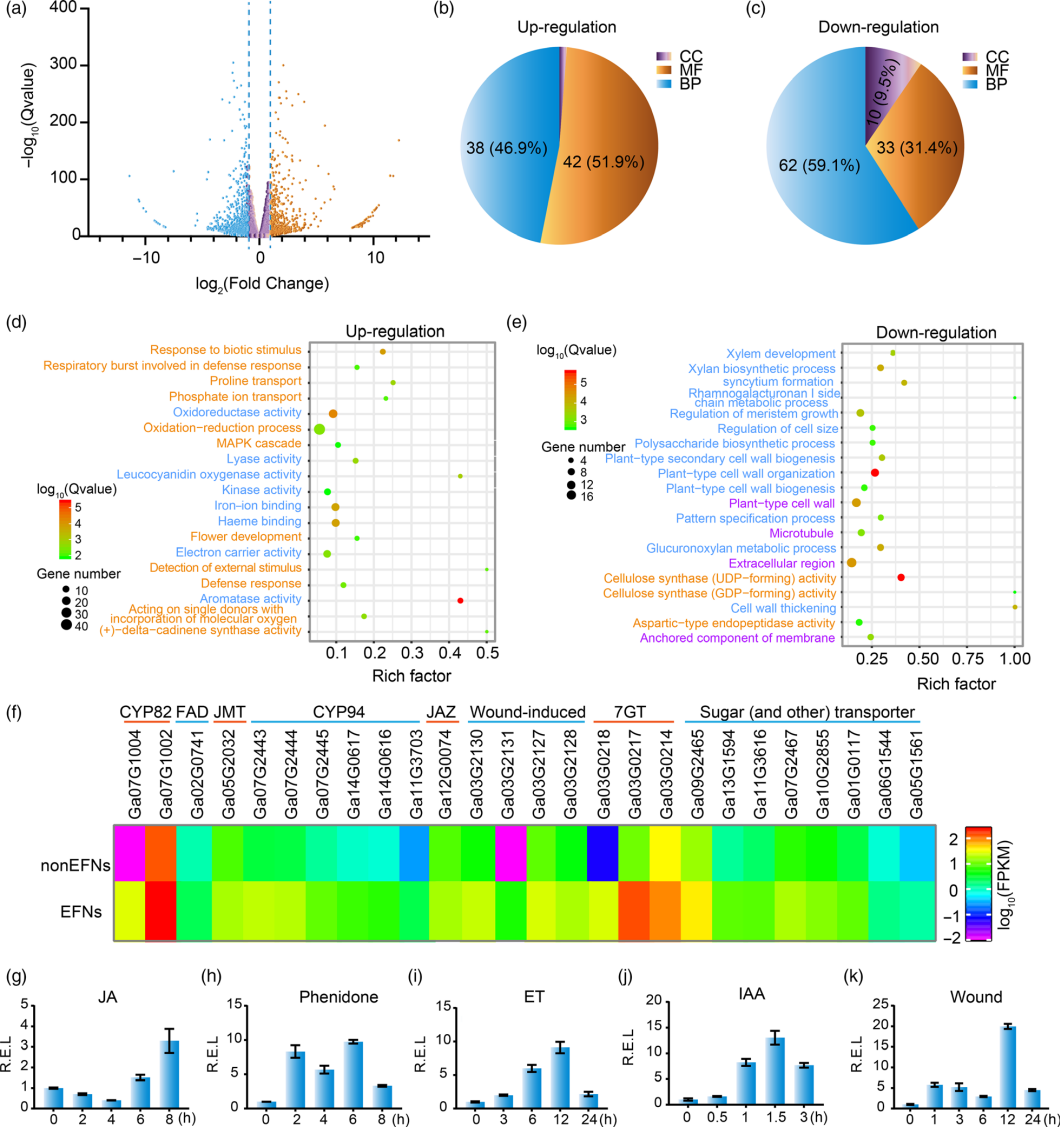
mRNA and qPCR analysis indicating hormones and cell wall‐related genes involved in nectary development. (a) Volcano plot of differentially expressed genes between nectary and nectariless leaf midribs. (b‐c) The frequency of GO terms distribution in biology process (BP), cellular components (CC) and molecular function (MF) in up‐ and down‐regulated DEGs, respectively. (d) The top 20 pathways enriched with up‐regulated genes. (e) The top 20 pathways enriched with down‐regulated genes. Names marked light blue, orange and purple represent BP, MF and CC terms, respectively. (f) DEGs involved in JA synthesis and metabolism, wound‐inducing, flavonoid synthesis and sugar transport. (g‐k) *GaNEC1* expression patterns under 1 mmol/L JA (g), 1 mmol/L phenidone (h), 5 mg/L ethephon, wound and 50 mg/L IAA treatments. The data in (g‐k) represent the means ± SD from three independent experiments.

To gain insight into the network underlying nectary development, mRNA‐Seq of EFN and non‐EFN foliar mid‐veins was performed to compare their gene expression. On the basis of comparisons yielding an adjusted *P*‐value ≤ 0.01 and an absolute value of fold change ≥ 2.0, 2393 total genes were identified as differentially expressed genes (DEGs), including 1009 up‐regulated genes and 1384 down‐regulated genes (Figure 5a).

The DEGs were mapped to GO terms using the R package ‘goseq’. Of the up‐regulated genes, 38 (46.9%), 42 (51.9%) and 1 (1.2%) gene terms were classified as biological process (BP), cellular components (CC) and molecular function (MF), respectively (Figure 5b). The down‐regulated genes were comprised of 62, 33 and 10 pathways classified as BP, MF and CC terms, respectively (Figure 5c). We found that genes related to cell wall thickening, cell wall organization, regulation of cell size and regulation of secondary cell wall biogenesis were down‐regulated in EFN cells (Figure 5e and S5), consistent with differences in cell morphology and intracellular components for EFN and non‐EFN cells as shown in Figure 2i‐p. The up‐regulated genes included genes related to aromatase activity, defence response, amino acid transport, carbohydrate transport, UDP‐glucosyl transferase activity, alkaloid biosynthesis and signalling. Strikingly, JA‐related genes, like fatty acid desaturase (*FAD*), jasmonic aicd carboxyl methyltransferase (*JMT*), jasmonic acid‐hypersensitive (*CYP82*) and jasmonoyl‐isoleucine‐12‐hydroxylase (*CYP94*), were up‐regulated, suggesting that the JA pathway is involved in nectary formation and nectar secretion in cotton. Wounding‐induced genes, flavonoid 7‐O‐glucosyltransferase (*7‐GT*) and sugar transporters were also up‐regulated (Figure 5d and f).

KEEG analysis showed enrichment of up‐regulated genes for genes related to MAPK signalling, pentose and glucuronate interconversions, terpenoid biosynthesis, plant hormone signal transduction, plant–pathogen interaction and flavone and flavonol biosynthesis, all of which may contribute to nectar secretion and EFN function (Figure S6‐7).

A previous study indicated that plant hormones and leaf damage are involved in nectary development (Roy *et al.*, 2017). To test if *GaNEC1* was induced by leaf damage and hormones, G*aNEC1* expression profiles were measured under treatments with JA, JA biosynthesis inhibitor phenidone, ET, IAA and wounding. Results showed that *GaNEC1* expression was induced under all treatments (Figure 5g‐k), indicating that *GaNEC1* is connected to multiple hormone signalling pathways and JA has dual regulatory effects on nectary development as the dual roles ethylene on fibre elongation (Li *et al.*, 2015).

### VIGS of *GaNEC1* resulted in nectary cell enlargement

**Figure 6:**
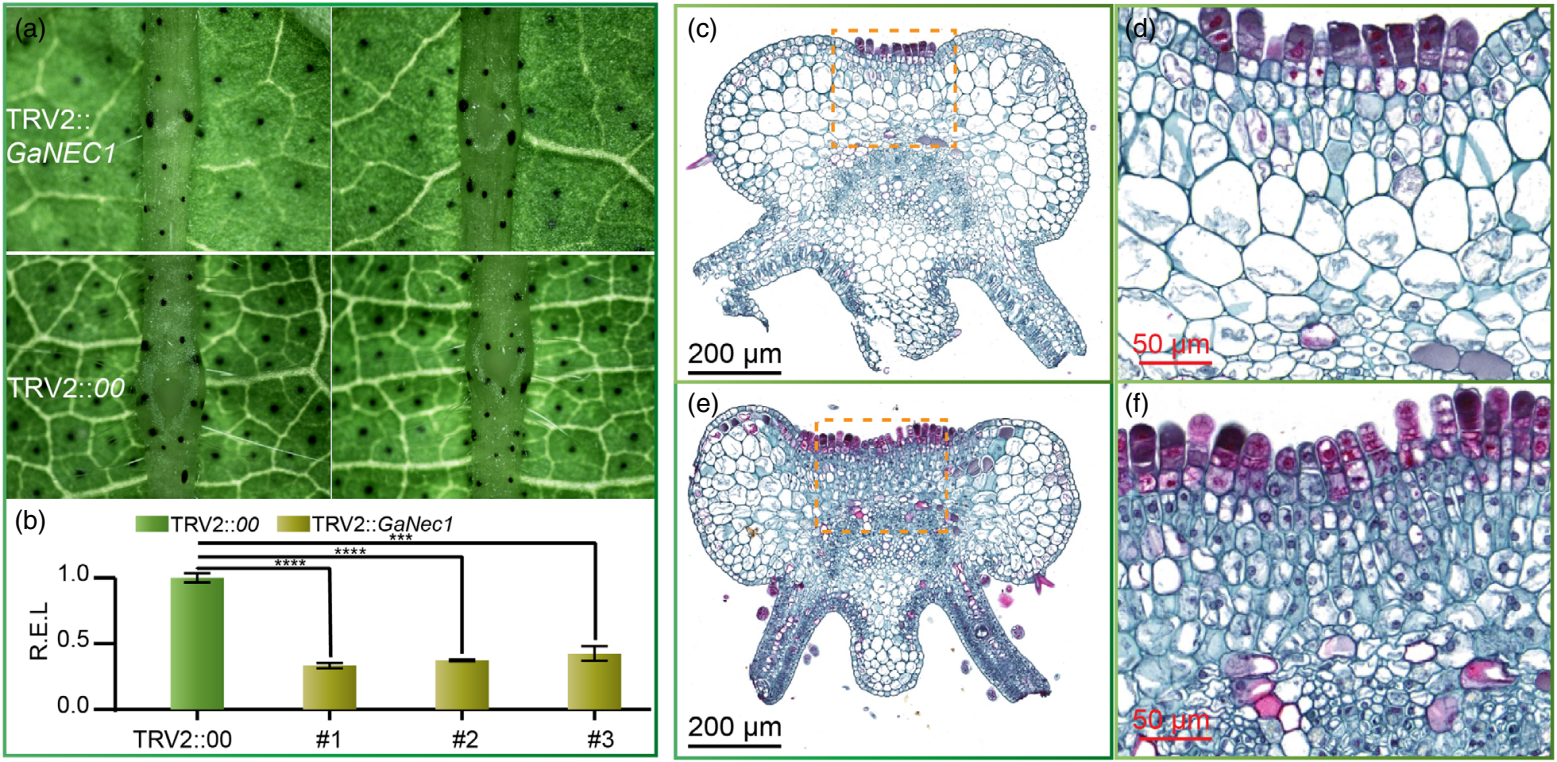
Silencing of *GaNEC1* in foliar nectary plants. (a) Phenotyping of GA0029 in *GaNEC1* silencing plants and blank vector infiltration plants. (b) *GaNEC1* transcript level in VIGS plants. Independent t‐tests indicated that there were significant differences among the TRV2::*00* and VIGS plants (#1, #2 and #3) at ∗∗∗*P* < 0.001, ∗∗∗∗*P* < 0.0001. Error bar represents SD of three independent experiments. (c) Cell morphology of VIGS plants. (d) Enlargement of the dotted line box in (c). (e) Cell morphology of TRV2::*00* infiltration plants. (f) Enlargement of the dotted line box in (e). [Correction added on 14 April 2020, after first online publication. Figure 6 was previously mislabelled. This has been amended in the current version of the article.]

To further test the gene function of *GaNEC1*, the 3’ segment of *GaNEC1* from GA0029 was cloned into TRV2::*GaNEC1* to knock down the expression of *GaNEC1* in foliar nectary accession GA0029 via virus‐induced gene silencing (VIGS) (Gong *et al.*, 2017). At 11 days postinfiltration, true leaves showed smaller nectaries in the midrib (Figure 6a). The qPCR results showed that *GaNEC1* expression was significantly depressed in the nectary midrib of VIGS leaves compared to that in leaves infiltrated with a blank TRV2::*00* vector, indicating that *GaNEC1* was efficiently silenced in VIGS lines (Figure 6b). We next used histochemical visualization to inspect the nectary structure. When compared with TRV2::*00* infiltration plants, VIGS plants display shallower nectary tissue grooves and larger nectary parenchyma cells. In VIGS plants, the morphology of parenchyma cells beneath the epidermis returned to an oval shape, similar to adjacent nectary cells (Figure 6c). Since silencing of *GaNEC1* can induce morphological changes similar to those induced by the nectariless allele, *GaNEC1* may be the key gene controlling nectary formation and regulate the expression of cell size and cell wall synthesis related genes that were identified in our mRNA‐seq analysis (Figure 6c). Unfortunately, a transgenic system is not currently available for *G. aboreum,* so it is not possible to test this hypothesis by with a nectariless mutant (containing the 15 bp InDels) created via genomic editing.

### Composition of cotton nectar

**Figure 7:**
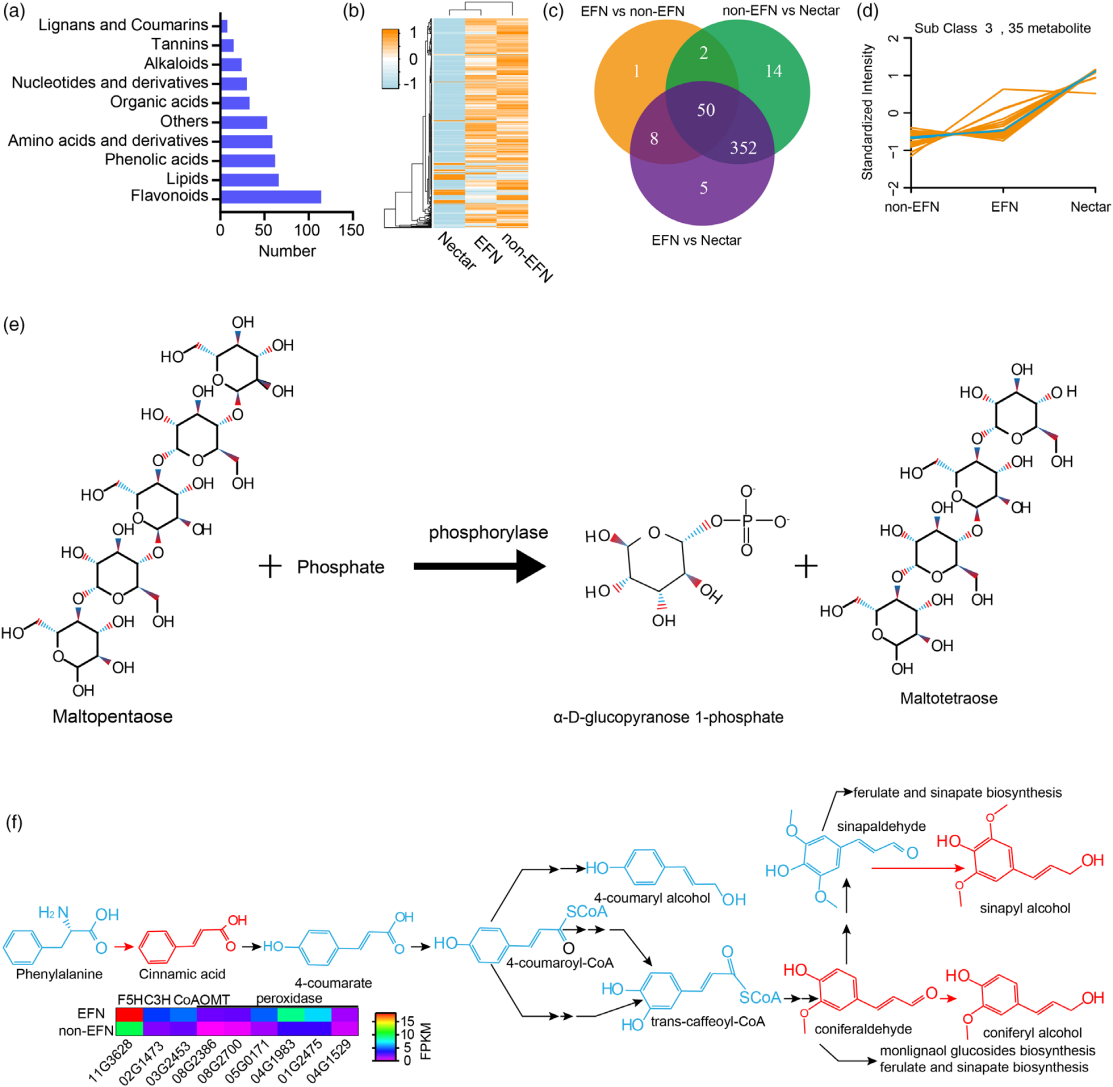
Components analysis of nectar. (a) The number of detectable metabolites in each group. (b) Clustering on the basis of metabolite components and contents. (c) Venn plots for differentiated metabolites between paired conditions. (d) The metabolites enriched in nectar compared with those in nectary or nectariless midrib. (e) The maltopentaose biosynthesis in plants. (f) Metabolites involved in the phenylpropanoid biosynthesis pathway are enriched in nectar.

Nectar is a complex mixture of diverse metabolites. Nectar is primarily composed of water, sugar and amino acids, but little is known about secondary nectar components. To identify key components in cotton nectar, we measured the metabolic composition of nectar, EFN foliar mid‐veins and non‐EFN foliar mid‐veins. The identified metabolites were divided into 10 classes, including expected sugars (divided into other groups here) and amino acids and 8 other classes: alkaloids, flavonoids, lipids, lignans and coumarins, nucleotides and derivatives, organic acids, phenolic acids and tannins (Figure 7a and Table S5). In general, the identified metabolites in nectar differed from those identified in EFN or non‐EFN foliar mid‐veins, and the amounts of most nectar metabolites were depleted in comparison with those from EFN or non‐EFN foliar mid‐veins (Figure 7b). More than 86% of the metabolites were at similar levels in EFN and non‐EFN foliar mid‐veins (Figure 7c). Our analysis identified 156 metabolites as enriched components of the nectar mixture (Table S5). The ten most abundant metabolites are classified as follows: two lipids (myristic acid and elaidic acid), three ‘others’ (sucrose, isomaltulose, and melibiose), two flavonoids (cyanidin 3‐o‐galactoside and gallocatechin 3‐O‐gallate), one organic acid (citric acid), one phenolic acid (coniferyl alcohol) and one nucleotide (adenosine). Of the 17 amino acids detected in nectar, three amino acids (L‐proline, L‐methionine and L‐glutamine) were the most abundant, and amino acids derivative that are toxic to pests were present (Roy *et al.*, 2017).

Kmeans method clustering divided all detected metabolites into 6 groups (Figure S8). The levels of metabolites from subclass 3 were higher in nectar than those in EFN and non‐EFNs veins. The metabolites (35 kinds) that were abundant in nectar may serve important functions (Figure 6d). Five sweet metabolites that were more abundant in nectar than EFN or non‐EFN veins (isomaltulose, D‐(+)‐sucrose, melibiose, D‐(+)‐melezitose and maltotetraose) could be toxic to nectar robbers that cannot digest these sugars (Figure S9) (Roy *et al.*, 2017). Maltotetraose was highly enriched in nectar (~21 000 times its levels in EFNs and non‐EFNs veins), which could increase the viscosity of nectar.

Phenolic acids metabolites (cinnamic acid, sinapyl alcohol and coniferyl alcohol) involved in phenylpropanoid biosynthesis are enriched in the nectar, which could inhibit herbivore damage to plants and enhance plants resistance to disease (García‐Lara *et al.*, 2004; Maher *et al.*, 1994; Santiago *et al.*, 2013). Phenylpropanoid biosynthesis is very important for lignin biosynthesis because lignin is a three‐dimensional polymer of phenylpropanoid alcohols. The expression of key phenylpropanoid biosynthesis enzymes, including *Ga03G2453* (caffeoyl‐CoA 5‐O‐methyltransferase), *Ga11G3628* (ferulate 5‐hydroxylase), and *Ga02G1473* (p‐coumarate 3‐hydroxylase), was up‐regulated in EFN veins (Figure 7f). Angiosperm lignification consumes both sinapyl alcohol and coniferyl alcohol and is possibly catalysed by a peroxidase (Boerjan *et al.*, 2003). At least six peroxidases were identified as up‐regulated genes EFNs vs. non‐EFNs, indicating that nectary tissue could produce more cell wall lignification than non‐EFN tissues. Additional lignification was confirmed by phloroglucinol staining that showed that nectary tissue contained more lignin than non‐EFNs (Figure S10). This result is consistent with the previous finding with *vicia faba* that increased lignification enhanced the stiffness of nectary tissue cell walls (Gish *et al.*, 2016).

## Discussion

### Genetic control of nectary formation in Cotton

Glands and trichomes are considered to be direct resistance traits, and they evolved independently (Rudgers *et al.*, 2004). The nectary, considered an indirect resistance trait, was widely distributed on wild cotton foliar midribs (Figure 1). Because of the absence of a foliar nectary, *G. tomentosum*, a cotton native to Hawaii, has been used to identify the nectarilessness allele in cotton. An F_2_ population derived from *G. hirsutum* and *G. tomentosum* hybridization was used to identify nectariless QTLs, and the nectary: nectariless segregation ratio indicated that the nectary phenotype is controlled by a single gene locus. Gene mapping showed that this trait was located in a region of D12 that is 0.6 cM from the nearest locus NAU4925‐150, which was annotated as *NE2* (Hou *et al.*, 2013). Another study showed that the nectariless trait also mapped to D12 (Chr.26) and suggested that the loss‐of‐function mutation persisted because there were no disadvantages in the absence of native ants in Hawaii (Waghmare *et al.*, 2005). In addition to *NE2*, *NE1* is another gene that also has a primary effect on foliar nectary formation and confers smaller foliar nectaries. *NE1* and *NE2* have been assigned to homologous chromosomes A12 and D12 (Waghmare *et al.*, 2005). In a report on upland cotton, a foliar nectariless phenotype was controlled by 2 genes, and BNL1673 was linked to the nectariless trait (Li *et al.*, 2011). We used the BNL1673 sequence as a query to blast the TM‐1 (Li *et al.*, 2015; Yang *et al.*, 2019) and *G. arboreum* (Du *et al.*, 2018; Li *et al.*, 2014) genomic databases. We found that the best BNL1673 match mapped to 86.46 Mb on A12 (the same chromosome as *NE1*) of *G. hirsutum*, and the second best match mapped to D12 (Figure S11). It is interesting that when BNL1673 is aligned against *G. arboreum*, it perfectly matches with Chr12 at 20.07 Mb, very close (~90‐kb‐distance) to *GaNEC1*, the gene locus we identified in this study (Figure S12). This indicates that nectariless traits in *G. hirsutum* and *G. arobreum* may both be controlled by orthologs of *NE1.*

We found that the ratio of foliar nectariless accessions was higher in YZR and SC geographic groups, indicating that nectariless plants may better fit the agricultural environment of the Yangtze River basin and South China. Nectariless can be considered as a defence trait that can prevent pests from harming cotton (Henneberry *et al.*, 1977; Lukefahr *et al.*, 1966; Lukefahr *et al.*, 1965; Lukefahr and Rhyne, 1960; Meredith *et al.*, 1973; Schuster *et al.*, 1976; Thomson *et al.*, 1987; Wilson and Wilson, 1976). In the era *G. arboreum* became widely planted in China, there were no pesticides and it was important for varieties to have insect resistance, which may explain the large‐scale adoption of foliar nectariless varieties.

QTL analysis of nectary and nectariless F_2_ population pools derived from hybridization of GA0029 (foliar nectary) and GA0028 (foliar nectariless) parents identified a 500 kb region that overlaps with the GWAS region and *F*_ST_ sweep region. Fine mapping with a large population narrowed the region to a ~96 kb segment that contains three genes, of which only *GaNEC1* (*Ga12G1409*) contained ORF sequence differences (a 15 bp InDel and one SNP) between the two parents. *Ga12G1411* was not expressed, and no differences in relative expression between the two parents were found for *Ga12G1410* (Figure S4). Thus, we conclude that *GaNEC1* is the causal gene for the nectariless trait.

### Multiple functions of nectary components

Along with the flowering nectary, the cotton foliar nectary is also an important tissue for nectar secretion. The major function of flower nectar is to attract insect or vertebrate pollinators, and EFNs attracts ants, parasitoids and predators, while also acting as an indirect defence against herbivores for more than 100 families of plants (Wackers and Bonifay, 2004). In this study, more than 400 components of nectar were identified and classified into 10 groups. Carbohydrates and free amino acids are the most important floral nectar components for pollinator attraction. Because different animals have different nutrition preferences, the composition of nectar determines the spectrum of nectar consumers. Sugars are the primary source of carbohydrates for visitors. The main solutes identified in most nectars are varying ratios of sucrose, glucose and fructose. The sucrose‐to‐hexose ratio ranges from nearly all sucrose to all hexose, which is very important in plant–mutualist interactions (Roy *et al.*, 2017). A recently study showed that glucose and fructose were the predominant components in *G. hirsutum* nectars (Chatt *et al.*, 2019). Instead of fructose and glucose, which were not detected, *G. arboreum* nectar includes concentrated sucrose, supporting that the chemical composition of nectar varies widely between species (Adler, 2009). Sugars are much more concentrated than amino acids, but the latter can significantly affect the attractiveness of nectar because the amino acid composition has been shown to be the key resource basis for mutualisms (Roy *et al.*, 2017). In the present study, we also found that the sugar content was higher than amino acid content in cotton nectar. Though amino acids are much less concentrated than sugars, they have been detected in all nectars examined to date and serve as key sources of nitrogen for legitimate nectar visitors. They also provide flavour to nectar and affect plant–mutualist interactions (Heil, 2011; Roy *et al.*, 2017). Other compound classes are also associated with nectar attraction. Volatile organic compounds have been shown to influence pollinator attraction, and nectar odour is also considered to be a signal for attracting pollinators. In cotton, the extrafloral nectar has been shown to serve as a food source for parasitic wasps, and odours were essential for attraction (Rose *et al.*, 2006).

Another important function of nectar is to provide protection through nectarine and secondary metabolites. Several studies have observed microbial infection via nectary in diverse plant species, including cotton, bean, squash, apple, pear, aucuba, banana, pineapple, hawthorn and gourds (Roy *et al.*, 2017). A previously study showed that *acacia* EFNs contain glucanases and chitinases that protect the plant from fungal infestation (Gonzalez‐Teuber *et al.*, 2009). Phenolic acids are essential for maize defence against maize weevils, and susceptibility to weevils was negatively correlated with changes in diferulic acids, grain hardness, *trans*‐ferulic acid and *p*‐coumaric acid (CA) (García‐Lara *et al.*, 2004).

In this study, we found intermediate products (phenolic acids) of phenylpropanoid biosynthesis are enriched in nectar, indicating that the enrichment of phenylpropanoid biosynthesis could contribute to prevention of fungal pathogen infections. Further, the phenylpropanoid biosynthesis is the major pathway for lignin biosynthesis, and we found the lignification of leaf midribs in nectary tissue was higher than that of nectariless tissue. The additional lignin increases the hardness of leaf veins, which may alleviate injury from herbivores as previous reported (Gish *et al.*, 2016). The secondary metabolites also play important roles in the defence system (Schuler, 2011). The cotton foliar nectary secreted non‐protein amino acids, phenolics, alkaloids and glycosides that appeared to be toxic or unpalatable to nectar robbers (Figure 7a).

### Regulation of nectar development

Studies showed that JA, auxin (IAA) and gibberellin play important roles in nectary function (Aloni *et al.*, 2006; Wiesen *et al.*, 2016). Cell wall invertase, induced by JA, is required for FN secretion in Arabidopsis. Further, studies in *Brassica napus*, tobacco and *Arabidopsis* showed that JA synthesis played significant roles in nectar secretion, and restriction of JA synthesis resulted in mutants that do not secrete nectar. Moreover, the JA‐responsive transcription factor *NtMYB305* in tobacco, its ortholog *MYB21* in *Arabidopsis* and *SWEET9* are required for nectar synthesis. In the current study, we found JA‐related genes that are required for plant growth developments and immune function, and *FAD*, *JMT*, *CYP82* and *CYP94*, were up‐regulated in nectaries. *FAD* is an endoplasmic reticulum enzyme that is responsible for the synthesis of 18:3 fatty acids from phospholipids. A loss‐of‐function *fad3‐2fad7‐2fad8* mutant is deficient in the jasmonate precursor linolenic acid and contained negligible levels of jasmonate. Mutant plants showed highly mortality from *Bradysia impatiens* attack and that mortality could be alleviated by application of exogenous methyl jasmonate (McConn and Browse, 1996). A loss‐of‐function *CYP82C2* mutant decreased expression of JA‐inducible defence genes and reduced the resistance to the necrotrophic fungus *Botrytis cinerea* (Liu *et al.*, 2010). *CYP82G1* is responsible for synthesis of terpene volatiles, which play important roles in plant–organism interactions as defence compounds against herbivores (Lee *et al.*, 2010). *JMT*, encoding a S‐adenosyl‐L‐methionine: jasmonic acid carboxyl methyltransferase, catalyses the formation of methyl jasmonate from jasmonic acid (Seo *et al.*, 2001). *JMT* was significantly up‐regulated in bollworm‐infested cotton bolls (Kumar *et al.*, 2016). *CYB94* genes, encoding jasmonyl‐L 12‐hydroxylase, are integral components of the fungus‐induced jasmonate metabolic pathway and attenuate defence response to *Botrytis cinerea* infection (Aubert *et al.*, 2015). *CYP94B3*‐overexpression plants were susceptible to insect attack (Koo *et al.*, 2011). In cultivated cotton, many studies have shown that nectaries tend to attract insect pests that reduce yield (Henneberry *et al.*, 1977; Lukefahr *et al.*, 1966; Lukefahr *et al.*, 1965; Lukefahr and Rhyne, 1960; Meredith *et al.*, 1973; Schuster *et al.*, 1976; Thomson *et al.*, 1987; Wilson and Wilson, 1976). Six *CYP94B* orthologs are up‐regulated in nectaries, indicating that up‐regulation of *CYP94B* may contribute to susceptibility of nectaries to insect pests.

Connected to differentiated expression of JA‐related genes between nectary and nectariless samples, *GaNEC1* expression was also induced by JA, JA inhibitor phenidone, wounding, ET and IAA, indicating that *GaNEC1* regulates nectary development and nectar secretion though phytohormones and downstream response genes. Fuller understanding of the roles of hormones in nectary function will require additional studies.

In sum, this study makes substantial progress in understanding extrafloral nectaries, which are of significant ecological and agricultural importance, by identifying a causal gene via genetic mapping. The identification of *GaNEC1* will enable further understanding of plant–mutualism interaction and co‐evolution. The natural variation of alleles can be utilized to create nectariless mutants with increased pest resistance that would serve as an alternative to chemical insecticides, which often comes with health and environmental risks.

## Methods

### Plant materials

Wild cotton species leaves were collected from the National Wild Cotton Nursery, Sanya, China. We selected 27 wild species representing the A to D plus K chromosome groups and allotetraploid AD group. Detailed information about sample collection is presented in Table S1. Two cotton species, including *G. gossypiodes* (Ulbrich) Standley (D_6_) and allotetraploid *G. tomentosum* (AD_3_), did not have any nectaries on the midribs of leaves. The 215 *G. arboreum* accessions were same as those used in our previous study (Du *et al.*, 2018) and were collected from south China, Yangtze River and Yellow River regions. The *G. arboreum* population was planted in the experimental field of the Institute of Cotton Research (ICR) CAAS at Anyang (N36°03′, E114°29) in 2015 and 2016. Foliar nectaries were investigated in the field (Table S2). An F_2_ population was generated by self‐fertilization of the F_1_ population derived from hybridization of GA0028 (nectariless) and GA0029 (Figure S13). The F_2_ population was planted in the experimental field of ICR at Anyang for phenotyping. Materials for RNA‐seq and qPCR analysis were also grown in the experimental field at Anyang in 2018. The leaves were treated under 1 mmol/L JA, 1 mmol/L phenidone, 5 mg/L ethephon, 50 mg/L IAA.

### Histochemical visualization

All the samples were collected from plants after the first flower bloomed except for the VIGS plants. For VIGS experiment, the samples were collected at 4–5 true leaves stage. The leaf midrib of nectary sections and corresponding sections cut from the midrib of nectariless leaves were analysed. Samples were fixed in formalin: acetic acid: 70% ethanol (1:1:18, v/v/v). Then, samples were dehydrated in a graded ethanol/tert‐butanol series, embedded in paraffin and sectioned to 7 μm on a rotary microtome (Leica Instruments GmbH, Wetzlar, Germany) (Yang *et al.*, 2014). The paraffin sections were stained with safranin and observed under the Olympus BX53 microscope (Olympus, Japan).

### Sampling and sequencing

For bulked segregation analysis, fresh young leaves that were collected from individuals of two parents (GA0028 and GA0029), 30 F_2_ progeny with foliar nectaries and 30 F_2_ progeny without foliar nectariless, were immediately frozen in liquid nitrogen. Genomic DNA was extracted with a previously reported method (Gong *et al.*, 2018; Li *et al.*, 2019). Equal amounts of DNA from 30 foliar nectary or foliar nectariless progeny were mixed to generate two DNA pool samples. At least 5 μg of genomic DNA for each sample were used to build paired‐end sequencing libraries, with approximately 500 bp inserts, in accordance with vendor‐provided instructions (Illumina). Each sample was sequenced at ~30× coverage of the assembled genome with 150‐bp paired‐end reads for each sample from the Illumina HiSeq X‐ten platform. For mRNA‐seq, total RNA was extracted with a previously reported workflow (Qin *et al.*, 2018; Yang *et al.*, 2017; Yang *et al.*, 2018; Zhang *et al.*, 2019). The mRNA was used to synthesis cDNA for building paired‐end sequencing libraries. The sequence libraries were sequenced on Hiseq X‐ten platform with the PE150 strategy by following the manufacturer’s instruction. The adaptor and contaminated reads (bacterial, mitochondrial and viral sequences, etc.) were removed by alignment to the NCBI‐NR database. Lower quality reads were filtered as we previously described (Liu *et al.*, 2018).

### Population genetics and GWAS analysis

The fixation statistic *F*_ST_ was used to indicate population differentiation on the basis of the variance of allele frequencies between nectary and nectariless groups. The SNP cluster for *F*_ST_ analysis was the same as previously reported (Du *et al.*, 2018). The *F*_ST_ was calculated using VCFtools (v0.1.12b) based on a 50 kb window with a 10 kb step. The top 1‰ of *F*_ST_ values (~0.2) were selected as candidates of highly divergent regions for further analysis. Adjacent divergent regions were merged into single regions. We used a total of 1,425,003 high‐quality SNPs (MAF > 0.05, missing rate <20%) from 215 *G. arboreum* accessions to perform GWAS of foliar nectary with efficient mixed‐model association expedited (EMMAX) software (http://genetics.cs.ucla.edu/emmax/). The genome‐wide significance thresholds were evaluated with the formula *P* = 0.05/n (where n is the effective number of independent SNPs) (Li *et al.*, 2012). The P‐value threshold for significance in the *G. arboreum* population was ~1.0 × 10^−6^.

### QTL mapping of nectary on the basis of BSA

Clean reads from the two parental and two progeny pools were mapped to the *G. arboreum* reference genome (Du *et al.*, 2018) using BWA (v0.7.13). Duplicated reads were removed by SAMtools (Li *et al.*, 2009). InDel‐Realigner and BaseRecalibrator in the GATK3.8 were used to realign InDels, and HaplotypeCaller and CombineGVCFs set to default parameters were used to call SNPs (McKenna *et al.*, 2010). The SNP index was calculated for both nectary and nectariless bulk samples by measuring the proportion of reads containing SNPs that were identical to those in the nectariless parent (GA0028). The ∆SNP index was calculated as (SNP index of nectary pool) – (SNP index of nectary pool). The average ∆SNP index was calculated based a 1000 kb sliding window with a 10 kb step size. Statistical 99% confidence intervals of the ∆SNP index were calculated under the null hypothesis (no QTL) (Takagi *et al.*, 2013).

### Fine mapping of nectary

Based on the BSA result, we got a single signal on Chr12 indicating that the nectary trait is controlled by a single gene locus. We analysed SRR and InDels markers within the candidate region (Chr12:18.8 Mb to 21.1 Mb) to design 184 pairs of SSR primers and 34 pairs of InDels primers. Two parents and 10 randomly selected F_2_ individuals were used to test the primers ability to detect polymorphisms. Then a small population, consisting of 94 F_2_ individuals and two parents, were used to test the linkage between markers and phenotypes. Finally, the linked markers were used to calculate the recombination values in a large population consisting of 2385 F_2_ individuals. In accordance with the number of individuals with exchanged markers, the candidate region was narrowed to a ~100 kb segment that encodes for three genes. The PCR procedures were as followed: 94 °C, 5 min; 94 °C, 30 s, 54 °C, 30 s, 72°C, 30 s, 35 cycles; 94°C, 5 min. PCR products were genotyped by visualization with 14% polyacrylamide gel electrophoresis.

### RNA‐seq and qPCR analysis

Clean reads were isolated by removal of adaptors, low quality reads and contaminated reads. Clean reads were mapped against the *G. arboreum* reference genome (ICR, V2.0) by Hisat2 (Pertea *et al.*, 2016). Expression levels were calculated by StringTie and normalized by fragments per kilobase of transcript per million fragments mapped (FPKM). A subscript (prepDE.py) within StringTie was used to obtain the raw reads count for each encoding gene. Differentially expressed genes between the two groups were identified by the R package ‘EdgeR’ (Robinson *et al.*, 2010). Benjamini and Hochberg’s approach was used to adjust the *P*‐value for the false discovery rate. Genes with an adjusted *P*‐value < 0.05 and absolute fold change value >2 were designated as differentially expressed genes. The GO enrichment was performed by the R package ‘go.seq’. The petal, flowering bud, stamen, fibre (10 DPA), root, stem cotyledon, hypocotyl, leaf and foliar nectary were harvested for qPCR analysis. The total RNA was extracted using a previous workflow (Qin *et al.*, 2018; Yang *et al.*, 2017; Yang *et al.*, 2018; Zhang *et al.*, 2019). The cDNA was synthesized using PrimeScript RT reagent Kit with gDNA Eraser (Takara, China). The *Gahistone3* (Cotton_A_11188) gene was used as an internal reference. PCR reactions were performed with an ABI 7900 real‐time PCR system (Applied Biosystems) using the SYBR Premix Ex Taq kit (Takara, China) with the manufacturer’s protocols. A three‐step method was used for PCR amplification with parameters as follows: 40 cycles of 95°C for 30 s, 95°C for 5 s and 95 °C for 30 s. The dissociation curves for each reaction were checked, and the relative expression levels of each target gene were calculated using the 2^−△△CT^ method. Three replicates were conducted for each sample. Primers used in this study were designed by Preprimer 3.0 program (Table S4).

### Virus‐induced gene silencing in *G. arbourem*

We used the TRV vector (i.e. TRV1 vectors TRV2) for VIGS, with TRV2:*:CHLI* (encoding magnesium chelatase subunit I) as a positive control. We cloned a 208 bp *GaNEC1* fragment from GA0029 cDNA with the PYL156‐1409‐F/PYL156‐1409‐R primer pair (Table S4). PCR product was inserted in the TRV2 with *Xba I* and *Sac I* (BioLabs) sites. The vector infiltration of cotton and silencing efficiency check was done by following a previous workflow (Gong *et al.*, 2017).

### Metabolites analysis

Nectar was collected at times between 14:00 and 16:00 from the leaf midrib using a 2 μL pipette. The foliar nectary and corresponding parts of nectariless samples were cut from leaf midribs. Freeze‐dried nectary and nectariless samples were crushed using a mixer mill (MM 400, RETSCH). To extract metabolites, a 100 mg sample was added to a PE tube with 1.2 mL 70% aqueous methanol and incubated at 4 °C for about 16 h. Then, following centrifugation at 12 000 *g* for 10 min, the supernatant was filtered before UPLC‐MS/MS analysis. Sample extracts were analysed using an UPLC‐ESI‐MS/MS system (UPLC, Shimadzu; MS, Applied Biosynthesis, 6500 Q TRAP, Applied Biosynthesis). Analysis conditions and metabolite annotations were as previously described (Zhu *et al.*, 2018). The clustering heatmap was generated by the R package ‘gplots’.

## Conflict of interest statement

The authors have no conflicts of interest to declare.

## Author contributions

Z.E. Yang conceived and F Li designed the research. Z.E. Yang managed the project. Z.E. Yang performed genome sequencing and *F*_ST_ analysis. W. Qin prepared the populations for GWAS and fine mapping. W. Hu, Y. Jin and P. Wang prepared samples, performed phenotyping, and contributed to data analysis. Z.E. Yang designed the molecular experiments, and W. Hu and Q. Yan the molecular experiments and led interpretation of the molecular‐data analysis. Z.E. Yang, and W. Hu. prepared the figures and tables. Z.E. Yang and F. Li wrote and revised the manuscript.

## Supporting information


**Figure S1** Comparison of cotyledon nectaries between *G. hirsutum* and *G. arboreum*
**Figure S2** Multicellular structure of foliar nectary secretory trichomes of *G. arboreum*
**Figure S3** Sequence alignments of three candidate genes in QTL region
**Figure S4** The relative expression level of *Ga12G1410* in GA0028 and GA0029
**Figure S5** A DAG diagram for enriched GO terms related to BP in the down‐regulation genes
**Figure S6** Enriched pathways of up‐regulation genes through KEGG analysis
**Figure S7** Enriched pathway of down‐regulation genes through KEGG analysis
**Figure S8** Kmeans cluster of the metabolites
**Figure S9** The enriched sugars in nectar
**Figure S10** Phloroglucinol staining of EFN and non‐EFN midribs
**Figure S11** BNL1673 alignment against *G. hirsutum* reference genome
**Figure S12** BNL1673 alignment against *G. arboreum* reference genome
**Figure S13** Flowchart of F2 population construction

**Table S1** The panel information of investigated cotton species
**Table S2** The panel information of 215 accessions
**Table S3** Chi‐Square test for the ratio of nectary progenies to nectariless progenies
**Table S4** List of primers used in this study
**Table S5** The enriched components in the nectar mixture
