# Supplementary material for: Genetic and evolution analysis of extrafloral nectary in cotton
Source: Plant Biotechnol J. 2020 Mar 10;18(10):2081–95. doi: 10.1111/pbi.13366 (PMC7540171; doi:10.1111/pbi.13366)
Supplement: Supplementary file 1 — Figure S1 Comparison of cotyledon nectaries between G. hirsutum and G. arboreum Figure S2 Multicellular structure of foliar nectary secretory trichomes of G. arboreum Figure S3 Sequence alignments of three candidate genes in QTL region Figure S4 The relative expression level of Ga12G1410 in GA0028 and GA0029 Figure S5 A DAG diagram for enriched GO terms related to BP in the down‐regulation genes Figure S6 Enriched pathways of up‐regulation genes through KEGG analysis Figure S7 Enriched pathway of down‐regulation genes through KEGG analysis Figure S8 Kmeans cluster of the metabolites Figure S9 The enriched sugars in nectar Figure S10 Phloroglucinol staining of EFN and non‐EFN midribs Figure S11 BNL1673 alignment against G. hirsutum reference genome Figure S12 BNL1673 alignment against G. arboreum reference genome Figure S13 Flowchart of F2 population construction [file PBI-18-2081-s002.docx]

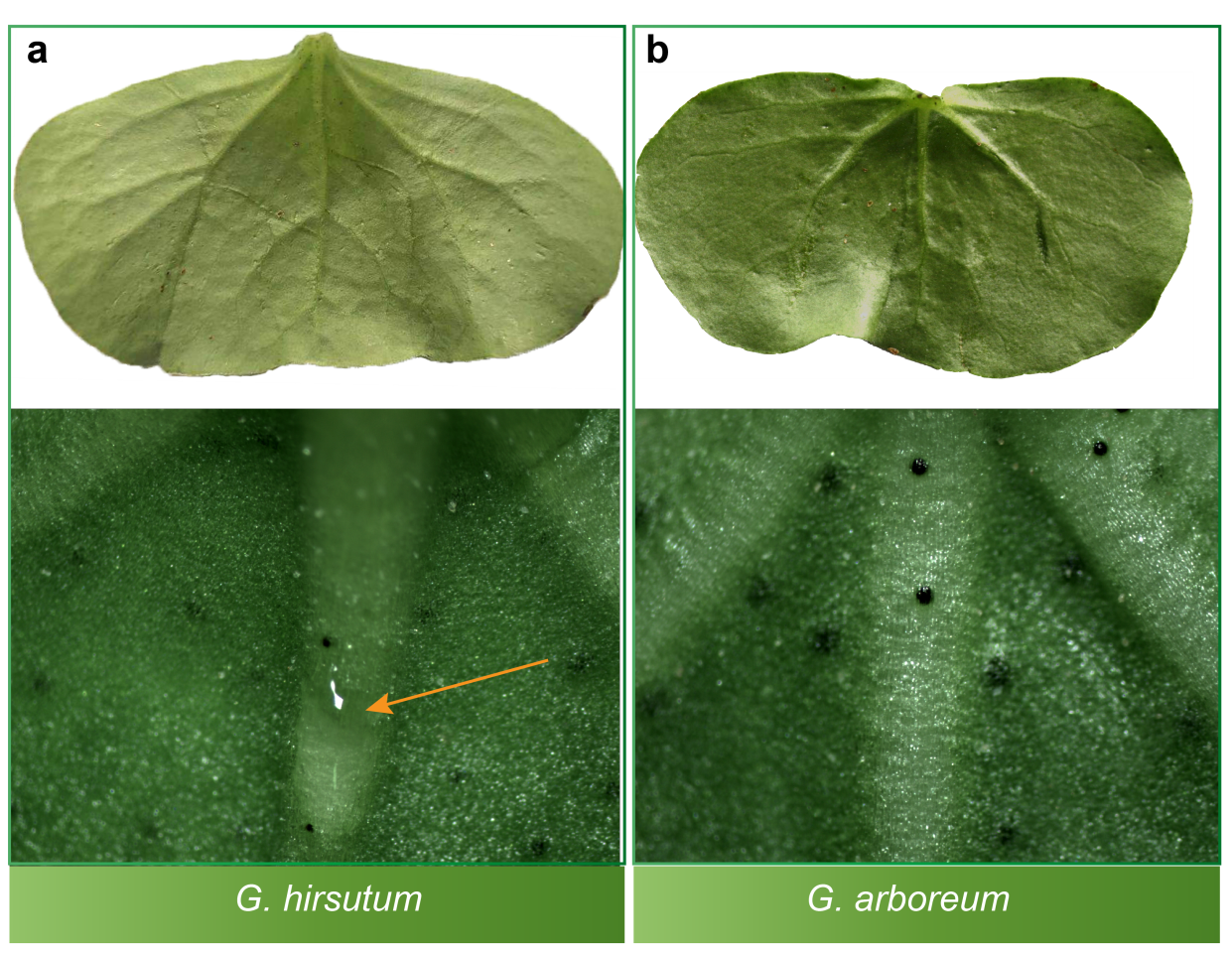


**Figure S1 Comparison of cotyledon nectaries between *G. hirsutum* and *G. arboreum****.* a，*G. hirsutum* acc. TM-1, bearing EFNs in the cotyledon essentially at emergence. b, *G. arboreum* didn’t grew out EFNs at the true leaf stage.


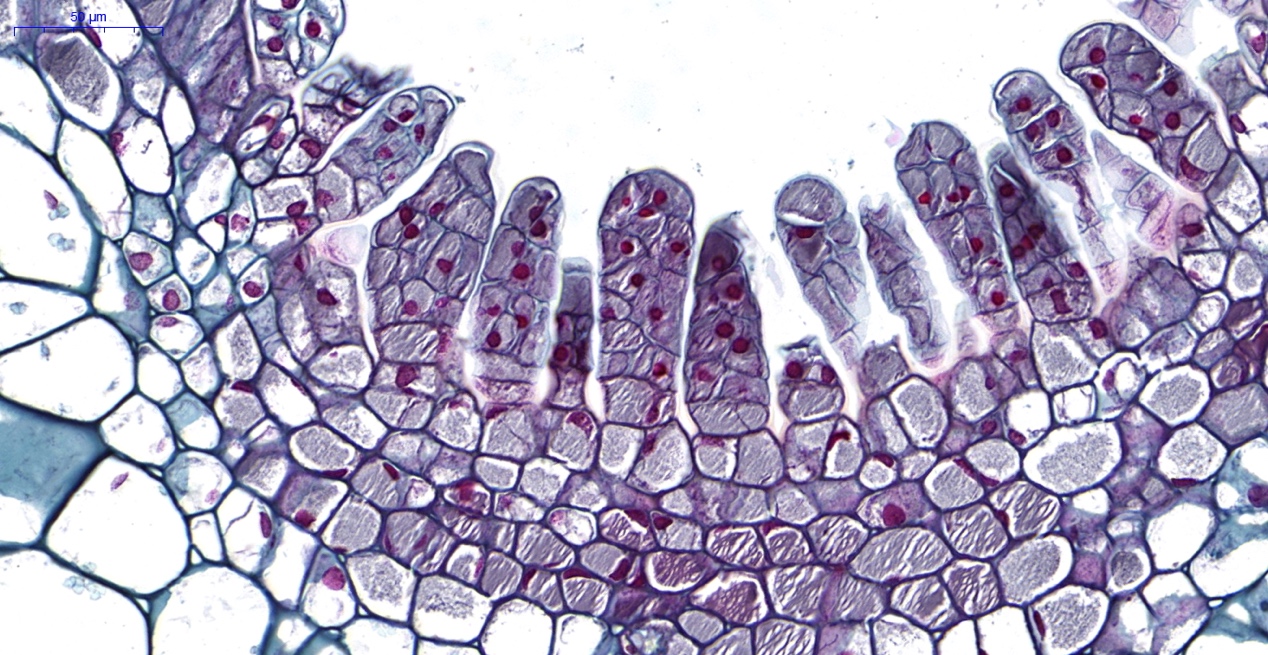


**Figure S2** **Multicellular structure of foliar nectary secretory trichomes of *G. arboreum*.**

**a**

GA0029 1 ATGCTCCACCCTAACCATAAACCTACCATCAAATTCCTCTGCAGTTACCGCGGTAAAATC
GA0028 1 ATGCTCCACCCTAACCATAAACCTACCATCAAATTCCTCTGCAGTTACCGCGGTAAAATC

GA0029 61 CTTCCTCGTTATCCTGACCGTAAACTCTGTTATCATGGTGGCGAAACCCGTGTACTCGTC
GA0028 61 CTTCCTCGTTATCCTGACCGTAAACTCTGTTATCATGGTGGCGAAACCCGTGTACTCGTC

GA0029 121 GTTGATCGCTCTATTTCCTTCTCCGAGTTGTCGTTGAAGATGGGGGAGATGTGTGGAACA
GA0028 121 GTTGATCGCTCTATTTCCTTCTCCGAGTTGTCGTTGAAGATGGGGGAGATGTGTGGAACA

GA0029 181 TCGGTGAGTTTACGTTGCCAGTTGCCAACAGAAGACTTGGACGCGCTGGTTTCGATTACT
GA0028 181 TCGGTGAGTTTACGTTGCCAGTTGCCAACAGAAGACTTGGACGCGCTGGTTTCGATTACT

GA0029 241 TCCGGTGAAGAACTCGCCTACCTCATCGAGGAATACGATCGGTTGGCATCGCCTGCATCC
GA0028 241 TCCGGTGAAGAACTCGCCTACCTCATCGAGGAATACGATCGGTTGGCATCGCCTGCATCC

GA0029 301 TTTTTAAAGATACGAGCTTTCCTTGGGATGCCAAAATCAACCACAAAATCAATTTCTCTA
GA0028 301 TTTTTAAAGATACGAGCTTTCCTTGGGATGCCAAAATCAACCACAAAATCAATTTCTCTA

GA0029 361 TCATCCTCGTCCTTAACGTTATCATCCACTTCATCTTCAAATTCATCTTCATCGTCATCA
GA0028 361 TCATCCTCGTCCTTAACGTTATCATCCACTTCATCTTCAAATTCATCTTCATCGTCATCA

GA0029 421 ACTCCCCGGTCTTCCTGCGTACGTCATATCCCCAGGACTCCCCCCGTCGCTTTCCCTCCT
GA0028 421 ACTCCCCGGTCTTCCTGCGTACGTCATATCCCCAGGACTCCCCCCGTCGCTTTCCCTCCT

GA0029 481 TGCTCCTCCAAGAAATCAACCACAAACAATATTCCTTGCTATGGTTATCTAGTTCATCAT
GA0028 481 TGCTCCTCCAAGAAATCAACCACAAACAATATTCCTTGCTATGGTTATCGAGTTCATCAT

GA0029 541 GGCAACCATATTCAGATTTACGTTAGAACTGGAAACACTGGCAATTGCAATAGCAGGAAC
GA0028 541 GGCAAC---------------GTTAGAACTGGAAACACTGGCAATTGCAATAGCAGGAAC

GA0029 601 CAGCATCATAAACTGTTTACCTATTTTGAATTGGCGAAGATCAAT
GA0028 586 CAGCATCATAAACTGTTTACCTATTTTGAATTGGCGAAGATCAAT

**b**
GA0028 1 GTCATAAAGAAGCAGCTCAATTCAGACATCGGAGTTTCCCTTATTATGACCAACTTACTG
GA0029 1 GTCATAAAGAAGCAGCTCAATTCAGACATCGGAGTTTCCCTTATTATGACCAACTTACTG

GA0028 61 CCATCTACGCAAAAGATTGAACCACTGGAAAAGATGCTCAAACAGCTGCTGATATTATTG
GA0029 61 CCATCTACGCAAAAGATTGAACCACTGGAAAAGATGCTCAAACAGCTGCTGATATTATTG

GA0028 121 AAGAAATAAATGCTGATGATGTAGCTGCTATAAATACTCATGAAGAAAGAAACGATTTCT
GA0029 121 AAGAAATAAATGCTGATGATGTAGCTGCTATAAATACTCATGAAGAAAGAAACGATTTCT

GA0028 181 ATGAATCTGAAGCTGATGTTTCTTTGGATGACATGGATCTGAAGCTGATGTTTCTTTGGA
GA0029 181 ATGAATCTGAAGCTGATGTTTCTTTGGATGACATGGATCTGAAGCTGATGTTTCTTTGGA

GA0028 241 TGACACAACCGCAACTAGAACCGCAACCAGCTAAAAACCAAGGTGATTCTGCATTTTCAA
GA0029 241 TGACACAACCGCAACTAGAACCGCAACCAGCTAAAAACCAAGGTGATTCTGCATTTTCAA

GA0028 301 AGAAGAAAAAAAAGATTTCTGATGCAAGTGATTTTTCTACTTCATTTAA
GA0029 301 AGAAGAAAAAAAAGATTTCTGATGCAAGTGATTTTTCTACTTCATTTAA

**c**

GA0028 1 ATGAAATTGAACTTAAGACTTAATTGCATTTATAAACTCAACTTTACCATTTCAATTAAA
GA0029 1 ATGAAATTGAACTTAAGACTTAATTGCATTTATAAACTCAACTTTACCATTTCAATTAAA

GA0028 61 GCCACATTTGATATTTATTTTAATTCTTTTTATAAATTTATTATATAATTTTTTTCCACA
GA0029 61 GCCACATTTGATATTTATTTTAATTCTTTTTATAAATTTATTATATAATTTTTTTCCACA

GA0028 121 CACAAGTGCCTCATAATAATTTTTTTTTTAATTTTGGATTTTGAAATATTTATTTTAGGT
GA0029 121 CACAAGTGCCTCATAATAATTTTTTTTTTAATTTTGGATTTTGAAATATTTATTTTAGGT

GA0028 181 TAAATAAATTATAAGAAATACACACTCTAAATGTGTATATATTGAAATACACATAGAATC
GA0029 181 TAAATAAATTATAAGAAATACACACTCTAAATGTGTATATATTGAAATACACATTGAATC

GA0028 241 TTAAATATACACTAAAAATATAAGCTCTAAAGCAAATTGAACAAATATATATTTAGTAAA
GA0029 241 TTAAATATACACTAAAAATATAAGCTCTAAAGCAAATTGAACAAATATATATTTAGTAAA

GA0028 301 ATTTTAAACTTAAAATATATCTTTAAAAATTAACTAAGGTTGACAATATTAAAATTATTT
GA0029 301 TTTTTAAACTTAAAATATATCTTTAAAAATTAACTAAGGTTGACAATATTAAAATTATTT

GA0028 361 TACAGTAAATTATTAAATTTAAAATATAATTTTAAATAAATACACGTTTTAATAACATTG

GA0029 361 TACAGTAAATTATTAAATTTAAAATATAATTTTAAATAAATACACGTTTTAATAACATTG

GA0028 421 ATTAAAATAAATTTATATTTTTCAAATATTTTTTTCAAATAATTAATTAATTCAAACAAA

GA0029 421 ATTAAAATAAATTTATATTTTTCAAATATTTTTTTCAAATAATTAATTAATTCAAACAAA

GA0028 481 ATTAAAATTATAAAATATTATAATATCAATACATACAAAAAAACTTGTGCATCGCACCAT

GA0029 481 ATTAAAATTATAAAATATTATAATATCAATACATACAAAAAAACTTGTGCATCGCACCAT

GA0028 541 ATTTCAAACTAGTTTATAATATATATATTTGAATTGTGTAACAAATAAAATTTATTCAAT

GA0029 541 ATTTCAAACTAGTTTATAATATATATATTTGAATTGTGTAACAAATAAAATTTATTCAAT

GA0028 601 TATTCACCTTCATTTTCCCAACATTGCATTAAAAAATTGTAAAAATTAGGAATCACTTGT

GA0029 601 TATTCACCTTCATTTTCCCAACATTGCATTAAAAAATTGTAAAAATTAGGAATCACTTGT

GA0028 661 AAATTTCTTGAATTACTAAGGTAATGTTTGGTTGGGTGTAATAGGAATACATTACAACCC

GA0029 661 AAATTTCTTGAATTACTAAGGTAATGTTTGGTTGGGTGTAATAGGAATACATTACAACCC

GA0028 721 AATGCAATGGGCCCACCACCAAACCAACGTTTGGTTGCCTTTAATGGCTATTACACGCTG

GA0029 721 AATGCAATGGGCCCACCACCAAACCAACGTTTGGTTGCCTTTAATGGCTATTACACGCTG

GA0028 781 AATGGAATCATCTTCTATTACAGTACGACCCGTAATAGCAATTCAGGGTCTTGGGGTCTG

GA0029 781 AATGGAATCATCTTCTATTACAGTACGACCCGTAATAGCAATTCAGGGTCTTAGGGTCTG

GA0028 841 ATTAGATATTCAAAGGCACGGGTAATAACATTTTTCCCTATTTTGGACCAAAATATCCTG

GA0029 841 ATTAGATATTCAAAGGCACGGGTAATAACATTTTTCCCTATTTTGGACCAAAATATCCTG

GA0028 901 TTTGTCATCTTCATCTTCATCGTTTTCACTTTCACACTTTAGACAATTTCTTCATAAACA

GA0029 901 TTTGTCATCTTCATCTTCATCGTTTTCACTTTCACACTTTAGACAATTTCTTCATAAACA

GA0028 961 TTTTTCCCAAAGTAAATGTTATAGAAATTGGATCCTTATCGACGACAAATCAAGCTTCAT

GA0029 961 TTTTTCCCAAAGTAAATGTTATAGAAATTGGATCCTTATCGACGACAAATCAAGCTTCAT

GA0028 1021 TCACCATCTTCATCTTCCTTCCGTTGAACCCATCTTCCTTCCGTTGAACCTCCTTATTTA

GA0029 1021 TCACCATCTTCATCTTCCTTCCGTTGAACCCATCTTCCTTCCGTTGAACCTCCTTATTTA

GA0028 1081 CGGTAAGTGATGGCCTTTGATTCCTATTTTTTTCCTCTAATTTTGTTAGTTAAAGTTCTT
GA0029 1081 CGGTAAGTGATGGCCTTTAATTCCTATTTTTTTCCTCTGATTTTGTTAGTTAAAGTTCTT

GA0028 1141 TTTCGGTCTCACTTTGATCCTTCTCAGTTTCTTCATTAGATCCTCATAATTTTTGTGTTC
GA0029 1141 TTTCGGTCTCACTTTGATCCTTCTCAGCTTATTCATTAGATCCTCATAATTTTTGTGTTC

GA0028 1201 TTTTTTCTGGGTTTCTAGAGTTTGATTTGGTAGCTGAATTGTTTTGGGTTTTAATGAATC
GA0029 1201 TTTTTTCTGGGTTTCTAGAGTTTGATTTGGTAGCTGAATTGTTTTGGGTTTTAATGAATC

GA0028 1261 AAAAGCTTCAACAAATTATTTTAAAGTGTTATTAGTTTGTTTGTGTGAATTTTGTAAATG
GA0029 1261 AAAAGCTTCAACAAATTATTTTAAAGTGTTATTAGTTTGTTTGTGTGAATTTTGTAAATG

GA0028 1321 TTTCATGCTTAATTTTTGTTTCTTAATTTTTATTTCATGATTTAAGTGGCTGTGCTATGG
GA0029 1321 TTTCATGCTTAATTTTTGTTTCTTAATTTTTATTTCATGATTTAAGTGGCTGTGCTATGG

GA0028 1381 TGTTACCCCTTTCCAGCTGCTGTTGTATCTACTACTTTGAAGCTTGCTAGTCACCACCGT
GA0029 1381 TGTTACCCCTTTCCAGCTGCTGTTGTATCTACTACTTTGAAGCTTGCTAGTCACCACCGT

GA0028 1441 CATCTCCAACTGTCGCTGTCAACGTCGAGTTCAGGTGAGGCTATACTACCATTTTTTTAC
GA0029 1441 CATCTCCAACTGTCGCTGTCAACGTCGAGTTCAGGTGAGGCTATACTACCATTTTTTTAC

GA0028 1501 AAACTTTTTGTTTGTTTGTTTGCGTAGCTTGGGTTGTAAAATGTTAACAAAGTTAAGCTG
GA0029 1501 AAACTTTTTGTTTGTTTGTTTGCGTAGCTTGGGTTGTAAAATGTTAACAAAGTTAAGCTG

GA0028 1561 TTTGTTCTGCTCTTTCTTTCATTCTTCTTGTTTTTCTTCCTTCACGCCACTCTCCCACCA
GA0029 1561 TTTGTTCTGCTCTTTCTTTCATTCTTCTTGTTTTTCTTCCTTCACGCCACTCTCCCACCA

GA0028 1621 CCGTTAGCTTATTATTGAGGTCATATTCCATCGATTACCCTAACCAATCAAGATCGCATG
GA0029 1621 CCGTTAGCTTCTTATTGAGGTCATATTCCATCGATTACCCTAACCAATCAAGATCGCATG

GA0028 1681 GCTACACCGTTGCCAATTCCACCTAAACTCCCTTGCAGCTGAAAGCTCTGACCTTTACTG
GA0029 1681 GCTACACCATTGCCAATTCCACCTAAACTCCCTTGCAGCTGAAAGCTCTGACCTTTATTG

GA0028 1741 CTTCGCTTGCCATGTTTACTTCTTTTCTCATTTTCCTCTTATTTTTCCTACCTCCGGCTT
GA0029 1741 CTTCGCTTGCCATGTTTACTTCTTTTCTCATTTTCCTCTTATTTTTCCTACCTCCGGCTT

GA0028 1801 TTCCTTTAAATTTGTGCTTCCTACTAAGCTTTTTTTTCTAATCTCTCATAGAAGGGTTAA
GA0029 1801 TTCCTTTAAATTTGTGCTTCCTACTAAGCTTTTTTTTCTAATCTCTCATAGAAGGGTTAA

GA0028 1861 GCATTACATTTGCTCAATCTATCCTGAGTATCTATGGATACTACTTGCGTCTGTTTGAAT
GA0029 1861 GTATTACATTTGCTCAATCTATCCTGAGTATCTATGGAGACTACTTGCGTCTGTTTGAAT

GA0028 1921 GCAAAAGCAAAATGGAATTCGATTTCATTTTGTTACGTTTGAAGTAAGAAATGTTAACTT
GA0029 1921 GCAAAAGCAAAATGGAATTCGATTTCATTTTGTTACGTTTGAAGTAAGAAATGTTAACTT

GA0028 1981 TGTTCATTTTCCGATTTTTTTGCTTTTTTATGTATTGTTTAATTATTTTGGAGTTGCACT
GA0029 1981 TGTTCATTTTCTGATTTTTTTGCTTTTTTATGTATTGTTTAATTATTTTGGAGTTGCACT

GA0028 2041 CACTGTAAATAAGTGCAAAAGTCTATAATAGAAATTTGATAAATATGTATTGTTCACTTT
GA0029 2041 CACTGTAAATAAGTGCAAAAGTCTATAATAGAAATTTGATAAATATGTATTGTTCACTTT

GA0028 2101 TCACAATCTTCTTAACTGCCACTTTTCACAATCTTCTTAACTGCTTATATGCTTTAGTGA
GA0029 2101 TCACAATCTTCTTAACTGCCACTTTTCACAATCTTCTTAACTGCTTATATGCTTTAGTGA

GA0028 2161 CCTACAATAAAAGAAAATTAACTAAAATAATTGTTCCCCTCTAAAACTACAAGTAGAGTT
GA0029 2161 CCTACAATAAAAGAAAATTAACTAAAATAATTGTTCCCCTCTAAAACTACAAGTAGAGTT

GA0028 2221 TCGTGATGTTGGCAATGGAAACAAAGTAAGTTTACGCTTCGGCCCTGAGGAGCTTGTTGA
GA0029 2221 TCGTGATGTTGGCAATGGAAACAAAGTAAGTTTACGCTTCGGCCCTGAGGAGCTTGTTGA

GA0028 2281 AAGTATGCATCTTAACTTGTTGAATTCTTTACCCAGTTTGCTTAAGTTAAAGTAATTGTG
GA0029 2281 AAGTATGCATCTTAACTTGTTGAATTCTTTACCCAGTTTGCTTAAGTTAAAGTAATTGTG

GA0028 2341 GGCTATGGTTATGGTATTGCATTGAGCTTGAACATTAGATATGGCTTAAGATTCTGCTAC
GA0029 2341 GGCTATGGTTATGGTATTGCATTGAGCTTGAACATTAGATATGGCTTAAGATTCTGCTAC

GA0028 2401 GAGATCAATGAGTTATATTCCTTTTACATTGTTTTGCAGGCCTAAGACATTTGACCAACT
GA0029 2401 GAGATCAATGAGTTATATTCCTTTTACATTGTTTTGCAGGCCTAAGACATTTGACCAACT

GA0028 2461 GCAAGTACTATTGGACTTATCGGCAAGTCTGCTACTTACCTAAAAGGTTGAAAAAATTAA
GA0029 2461 GCAAGTACTATTGGACTTATCGGCAAGTCTGCTACTTACCTAAAAGGTTGAAAAAATTAA

GA0028 2521 GACTGGACTTGTTGTGCACTTGGTTTGCTAGAAGTAATTTTAATTCTCCTTTTATAACTT
GA0029 2521 GACTGGACTTGTTGTGCACTTGGTTTGCTAGAAGTAATTTTAATTCTCCTTTTATAACTT

GA0028 2581 TTACCTCTGTTTTCTGTTATGGAGTTGACACTTCCATTACTACAGCCACTAATTATCCTT
GA0029 2581 TTACCTCTGTTTTCTGTTATGGAGTTGACACTTCCATTACTACAGCCACTAATTATCCTT

GA0028 2641 GCACTCATCTTCATCATCATTAAGATATTGCTATGCCATTAAAACTTGGATGTGATCTAT
GA0029 2641 GCACTCATCTTCATCATCATTAAGATATTGCTATGCCATTAAAACTTGGATGTGATCTAT

GA0028 2701 GGTTATGGTATTGCATTGAGCTTGAACATCAGATATGACTTATGGTATTGCAGTTTTTGT
GA0029 2701 GGTTATGGTATTGCATTGAGCTTGAACATCAGATATGACTTATGGTATTGCAGTTTTTGT

GA0028 2761 TGGAATAGTTTTTCTAAAGTGCGATTGAATATTAATCCCCAATGCAAAGTAAAAGTGTTT
GA0029 2761 TGGAATAGTTTTTCTAAAGTGCGATTGAATATTAATCCCCAATGCAAAGTAAAAGTGTTT

GA0028 2821 GTTTGGCCCTGTTATAGTGTTCATTTTGCCTTTGGTTTTTT-ACCAAGTTTATTTTTATT
GA0029 2821 GTTTGGCCCTGTTATAGTGTTCATTTTGCCTTTGGTTTTTTTACCAAGTTTATTTTTATT

GA0028 2880 TGAATTTTTTAGTTGGAACCATTATGCGTTAAATGTGTAGTGGTATATTATTTCCAACAC
GA0029 2881 TGAATTTTTTAGTTGGAACCATTATGCGTTAAATGTGTAGTGGTATATTATTTCCAACAC

GA0028 2940 TGGTTTATTATTTCCTTTTTAATTAATTTAATACAATACAAAAATGTTAATTGCCTCTAA
GA0029 2941 TGGTTTATTATTTCCTTTTTAATTAATTTAATACAATACAAAAATGTTAATTGCCTCTAA

GA0028 3000 ATCCCTGCCCCTTACCAACAAATAAACAATATTTACTTTTTAATAATGCATATTATCACC
GA0029 3001 ATCCCTGCCCCTTACCAACAAATAAACAATATTTACTTTTTAATAATGCATATTATCACC

GA0028 3060 TATTTTTATAAAAAAAAACTATAATGTTTTATATAATCACATTATGCAATATCATAATAC
GA0029 3061 TATTTTTATAAAAAAAAACTATAATGTTTTATATAATCACATTATGCAATATCATAATAC

GA0028 3120 CACGTTAGGTTTAA
GA0029 3121 CACGTTAGGTTTAA

**Figure S3** **Sequence alignments of three candidate genes in QTL region**. a, Sequence alignment of *Ga12G1409* between GA0028 and GA0029. A 15-bp InDel and one SNP were observed in the coding sequences. b, Sequence alignment of *Ga12G1410*. c, Sequence alignment of *Ga12G1411*. The exons are marked by red color background.

**
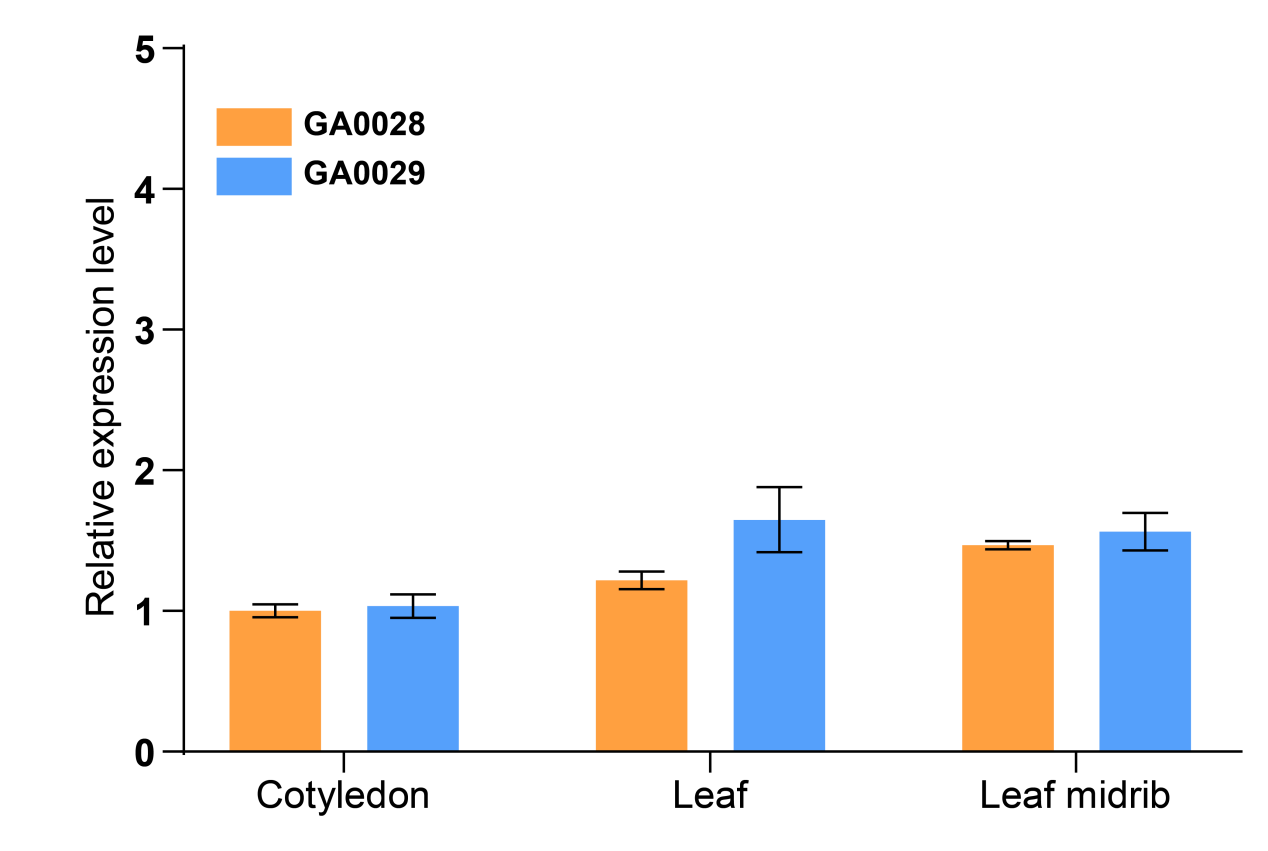
**

**Figure S4 The relative expression level of *Ga12G1410* in GA0028 and GA0029.**


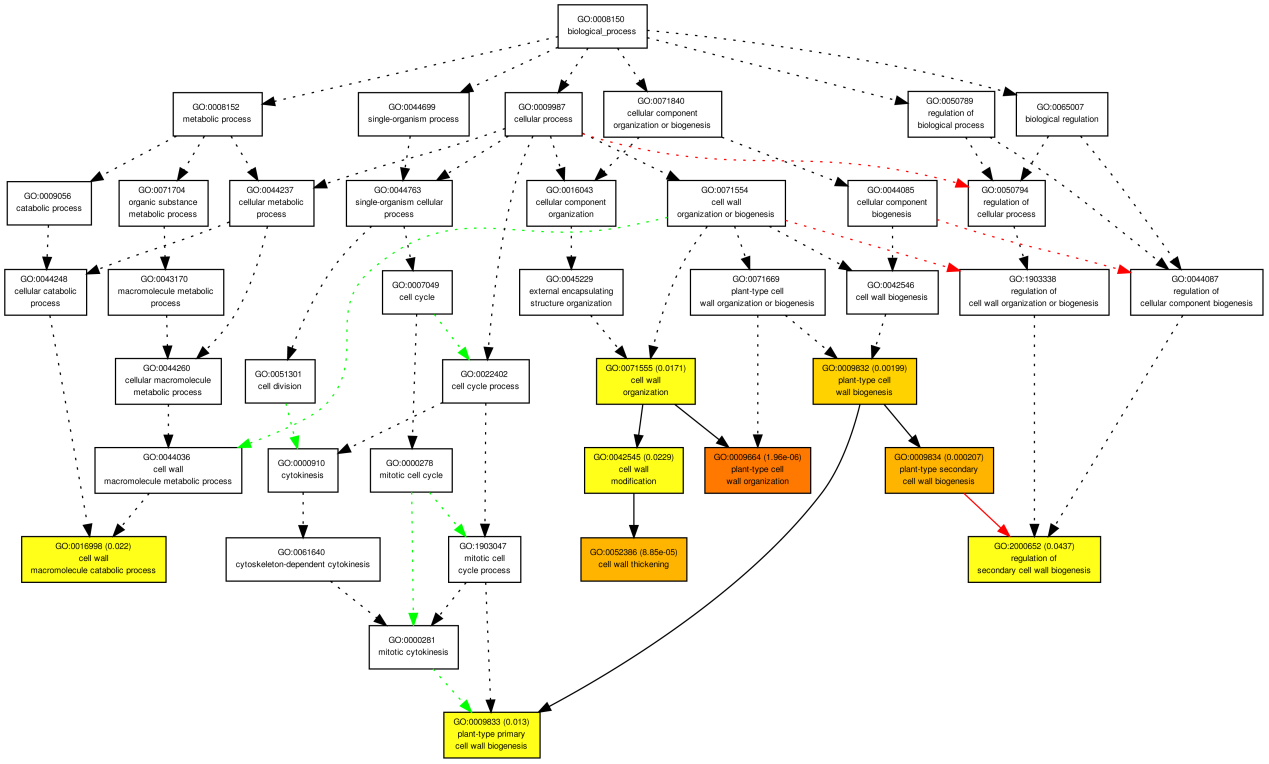


**Figure S5 A DAG diagram for enriched GO terms related to BP in the down-regulation genes**

**
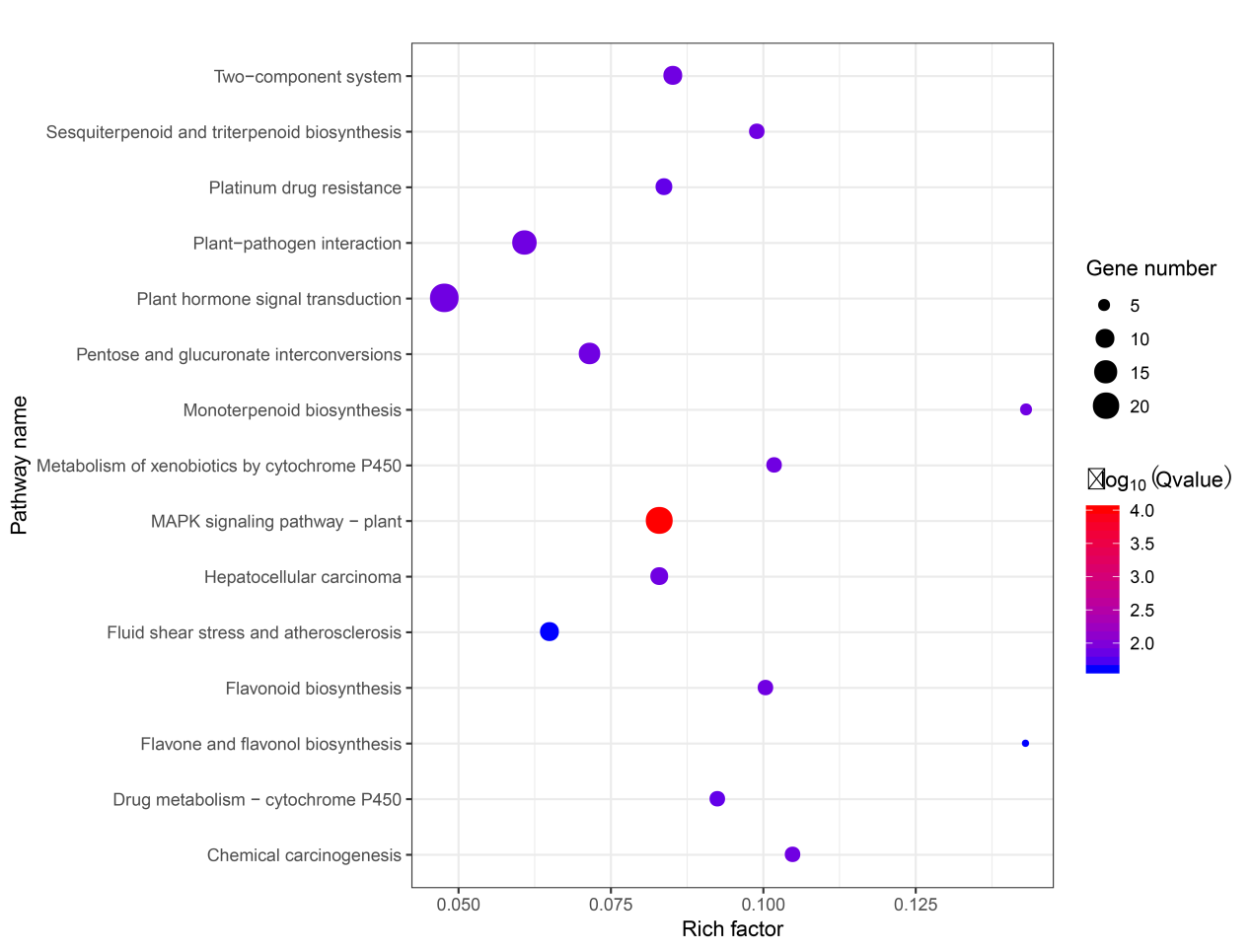
**

**Figure S6 Enriched pathways of up-regulation genes through KEGG analysis.**

**
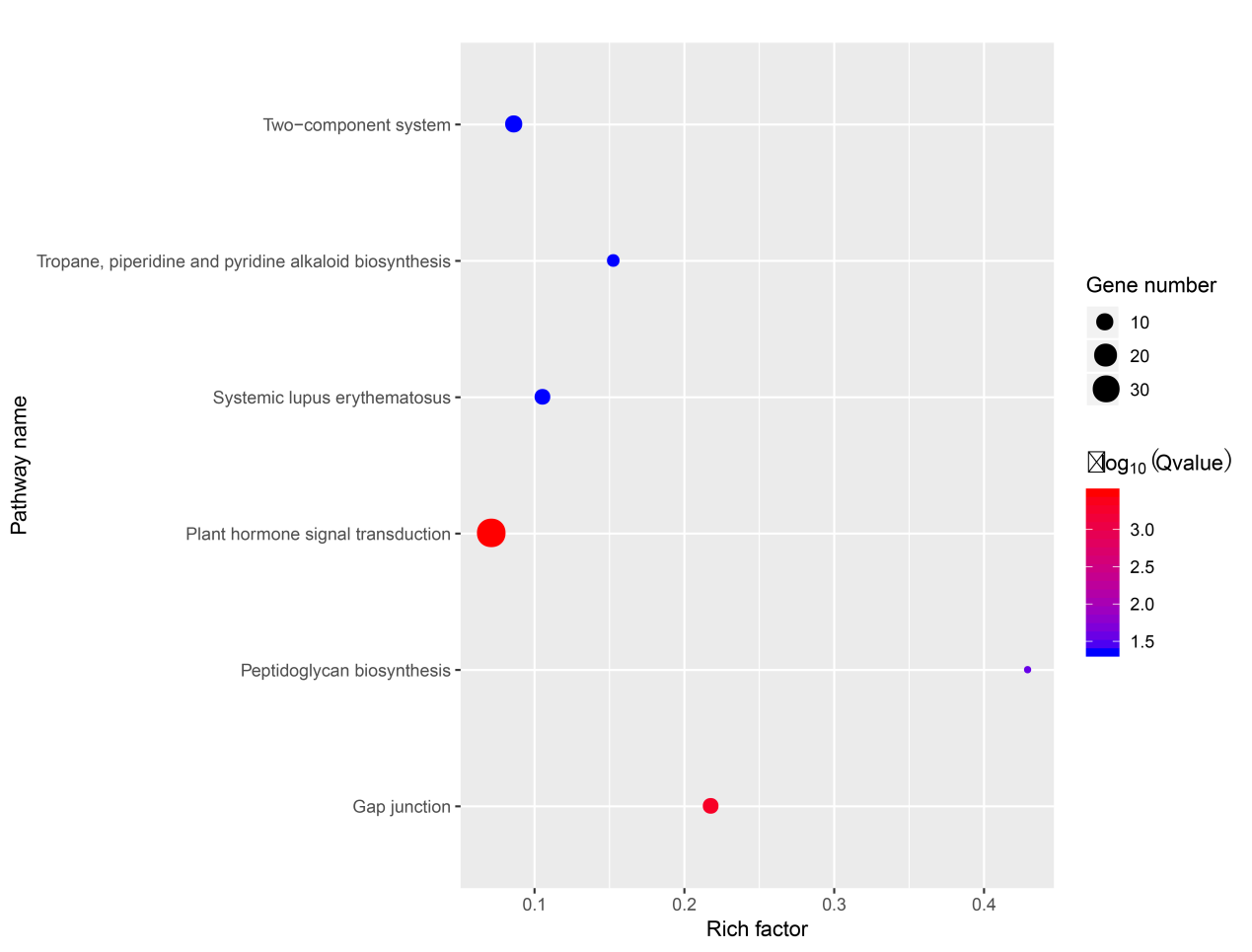
**

**Figure S7 Enriched pathway of down-regulation genes through KEGG analysis.**

**
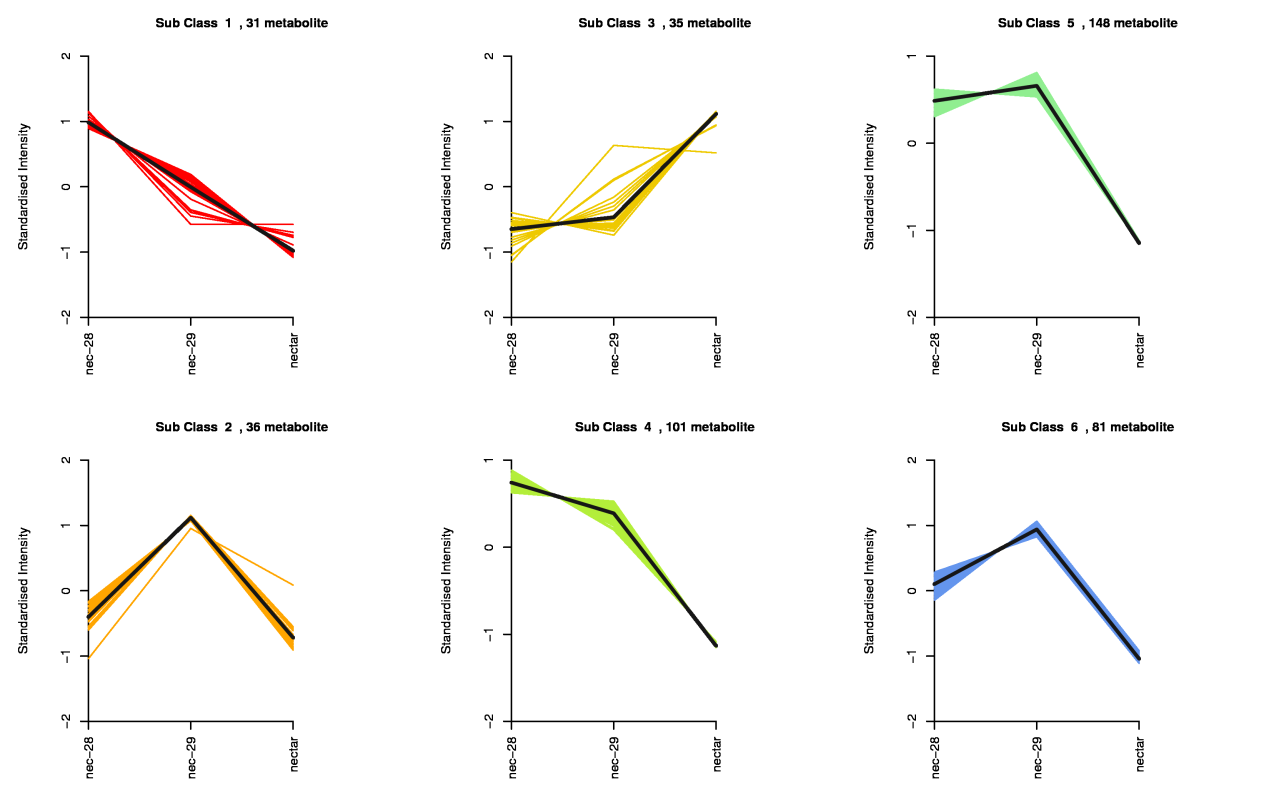
**

**Figure S8 Kmeans cluster of the metabolites.**


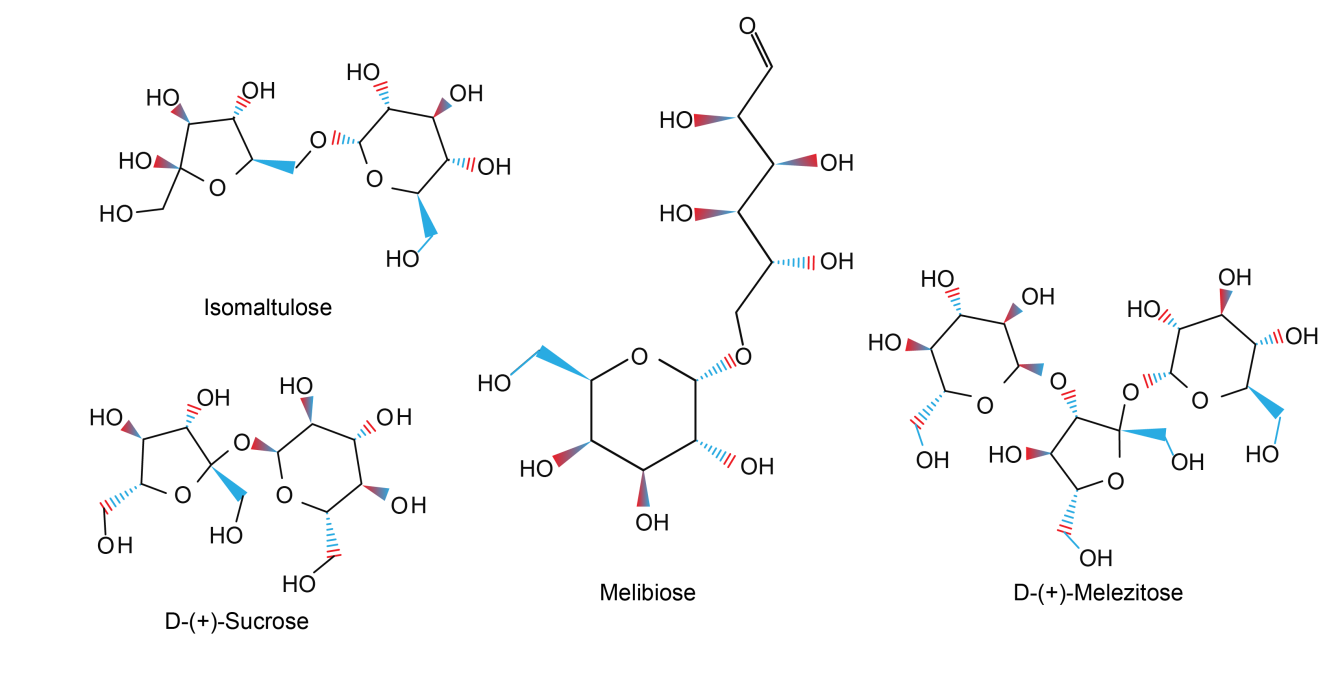


**Figure S9 The enriched sugars in nectar.**


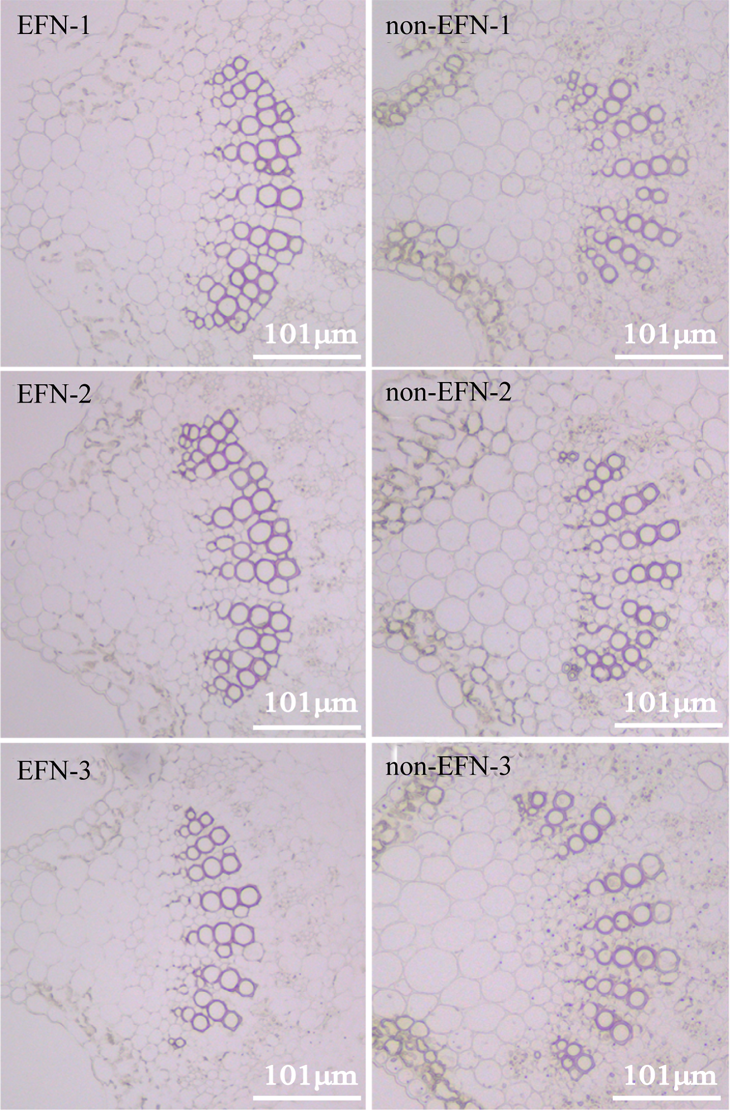


**Figure S10 Phloroglucinol staining of ENF- and non-EFN midribs.**


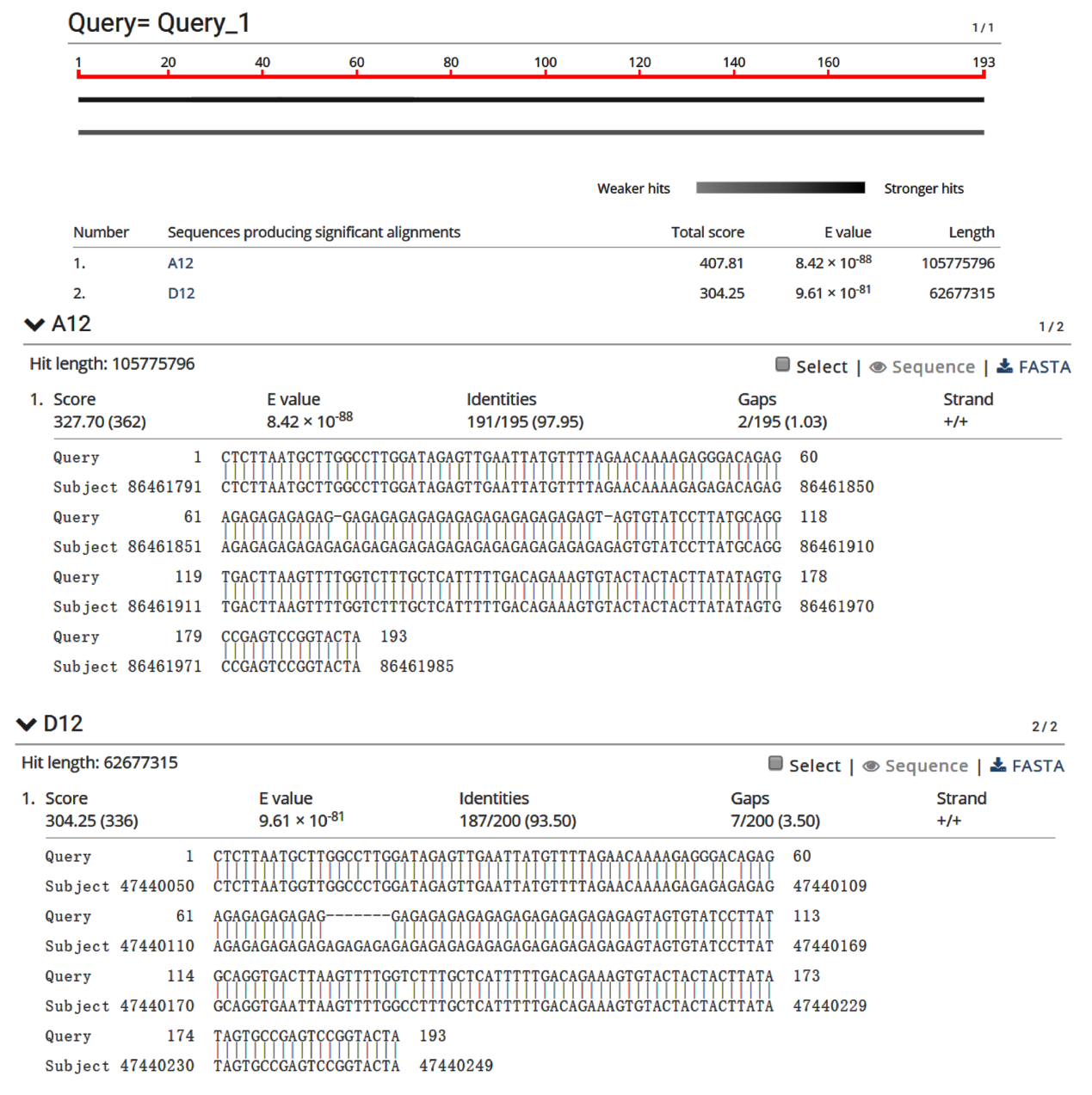


**Figure S11 BNL1673 alignment against *G. hirsutum* reference genome.**


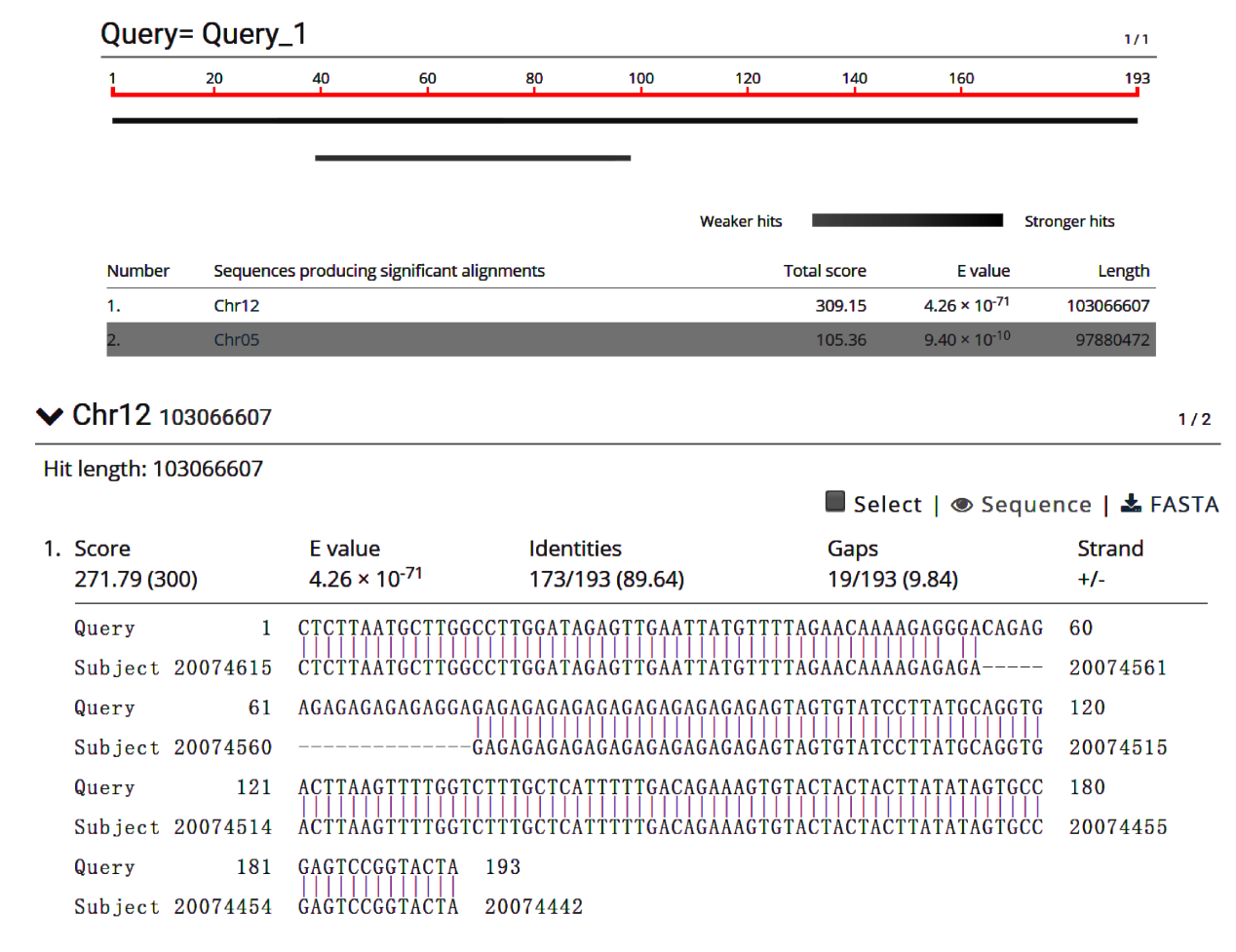


**Figure S12 BNL1673 alignment against *G. arboreum* reference genome.**

**
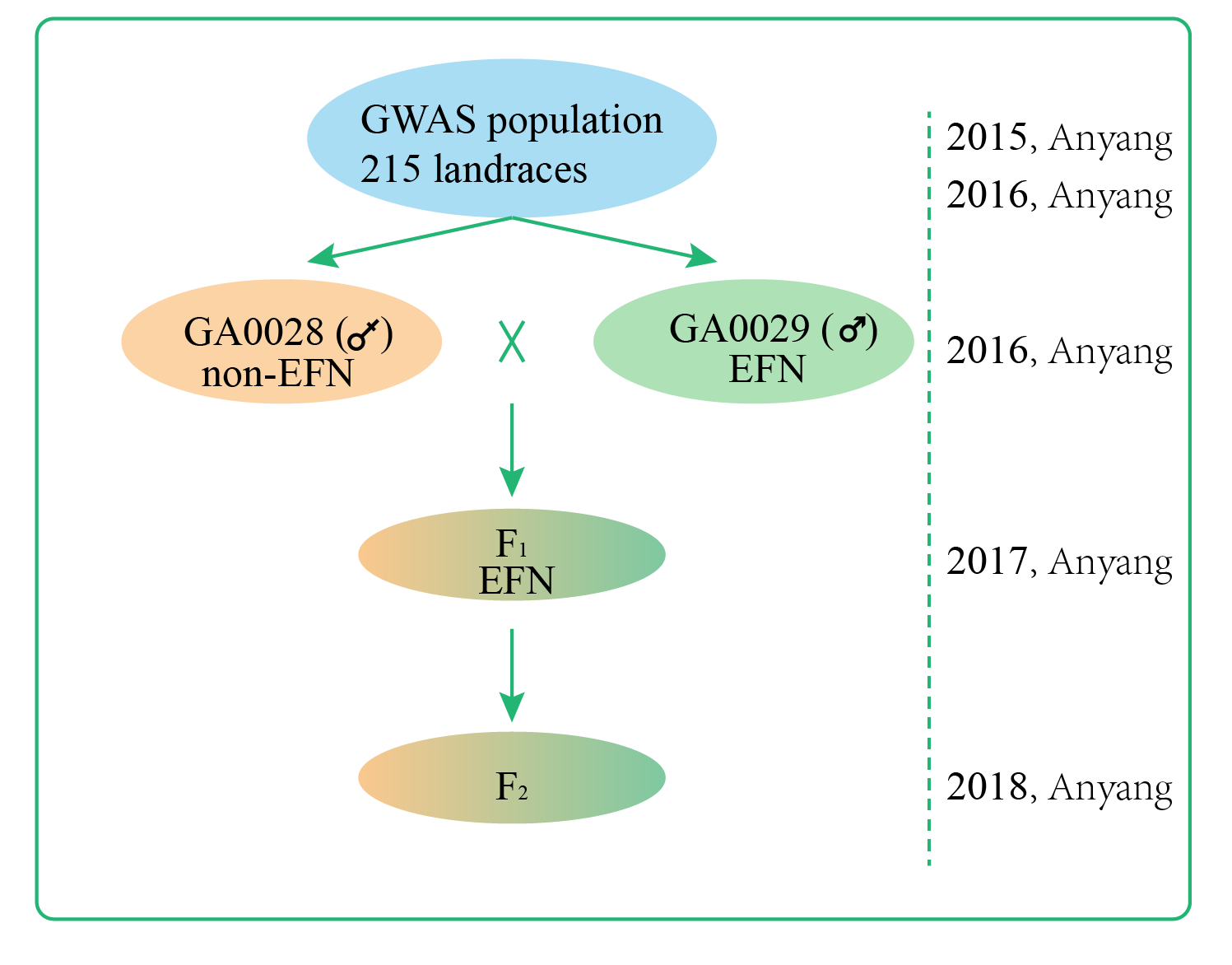
**

**Figure S13 Flowchart of F_2_ population construction.**
